# Guidance and Self‐Sorting of Active Swimmers: 3D Periodic Arrays Increase Persistence Length of Human Sperm Selecting for the Fittest

**DOI:** 10.1002/advs.201700531

**Published:** 2017-12-27

**Authors:** Thiruppathiraja Chinnasamy, James L. Kingsley, Fatih Inci, Paul J. Turek, Mitchell P. Rosen, Barry Behr, Erkan Tüzel, Utkan Demirci

**Affiliations:** ^1^ Bio‐Acoustic MEMS in Medicine (BAMM) Laboratory Canary Center at Stanford for Cancer Early Detection Department of Radiology Stanford School of Medicine Stanford University Palo Alto CA 94304 USA; ^2^ Department of Physics Worcester Polytechnic Institute Worcester MA 01609 USA; ^3^ The Turek Clinic San Francisco CA 94133 USA; ^4^ Department of OBGYN University of California San Francisco School of Medicine San Francisco CA 94158 USA; ^5^ Department of Obstetrics and Gynecology School of Medicine Stanford University Stanford CA 94305 USA; ^6^ Department of Electrical Engineering (by courtesy) Stanford University Stanford CA 94305 USA

**Keywords:** fertility, multiparticle collision dynamics, multiscale simulations, persistence length, sperm sorting

## Abstract

Male infertility is a reproductive disease, and existing clinical solutions for this condition often involve long and cumbersome sperm sorting methods, including preprocessing and centrifugation‐based steps. These methods also fall short when sorting for sperm free of reactive oxygen species, DNA damage, and epigenetic aberrations. Although several microfluidic platforms exist, they suffer from structural complexities, i.e., pumps or chemoattractants, setting insurmountable barriers to clinical adoption. Inspired by the natural filter‐like capabilities of the female reproductive tract for sperm selection, a model‐driven design, featuring pillar arrays that efficiently and noninvasively isolate highly motile and morphologically normal sperm, with lower epigenetic global methylation, from raw semen, is presented. The **S**imple **P**eriodic **AR**ray for **T**rapping **A**nd isolatio**N** (SPARTAN) created here modulates the directional persistence of sperm, increasing the spatial separation between progressive and nonprogressive motile sperm populations within an unprecedentedly short 10 min assay time. With over 99% motility of sorted sperm, a 5‐fold improvement in morphology, 3‐fold increase in nuclear maturity, and 2–4‐fold enhancement in DNA integrity, SPARTAN offers to standardize sperm selection while eliminating operator‐to‐operator variations, centrifugation, and flow. SPARTAN can also be applied in other areas, including conservation ecology, breeding of farm animals, and design of flagellar microrobots for diagnostics.

## 1 Introduction

Human infertility affects an estimated 15% of couples globally (about 50 million total), and nearly one‐third of these infertility cases are of male origin.^1^ Many such cases are treated with assisted reproduction technologies (ARTs),^2^, ^3^ most commonly using in vitro fertilization (IVF) and intracytoplasmic sperm injection (ICSI).^4^ During ART procedures, semen is traditionally processed with the density gradient centrifugation or swim‐up techniques.^5^, ^6^ With ICSI, individual sperm are then selected visually based on motility and morphology by a trained embryologist for injection to an egg.^7^, ^8^ However, other important sperm attributes that can affect ART outcomes, including sperm DNA–chromatin or chromosomal integrity, mutational, or methylome composition, and the presence of reactive oxygen species (ROS)^9^, ^10^, ^11^, ^12^, ^13^, ^14^, ^15^, ^16^, ^17^ are typically not considered. In particular, long periods of incubation, as well as shear stresses due to centrifugation, increase ROS accumulation. Additionally, sperm nuclear DNA damage is not only limited to infertile but also to subfertile patients. Even where the seminal parameters are normal, ejaculates showing sperm with high DNA damage have been reported.^10^, ^11^ Moreover, various epidemiological studies reporting the methylation status of imprinted genes of sperm from severely infertile men show a significant risk of imprinting disorders in offspring conceived by IVF and ICSI.^18^, ^19^, ^20^, ^21^, ^22^, ^23^, ^24^, ^25^, ^26^ These observations strongly argue that the sperm genome provides an epigenetically poised set of developmental genes that potentially have a crucial effect on embryological growth and development. Although the recent development of microfluidic devices,^27^, ^28^, ^29^, ^30^, ^31^ to some extent, addressed these needs around motility and DNA fragmentation,^32^ majority of these devices require chemoattractants and pumps to provide the required flow conditions, causing undesirable sample dilution and presenting structural barriers for implementation at the clinic. In addition, they have fallen short of sorting for epigenetic aberrations (**Table** **1**).^14^, ^33^, ^34^

**Table 1:** Comparison of different microfluidic sperm processing devices. N/A: Not available; Q/R: Qualitatively reported; VSL and VCL were indicated as a factor of change between inlet and outlet

Parameters		Flow or chemical‐based sorting			Passive sorting			
		Multiple channels^77^, ^78^	Single channel^79^	Chemotaxis based^80^	Other^81^, ^82^, ^83^, ^84^	Blank channel (BMC)^3^	Filter based (MSS)^6^	SPARTAN (this paper)
Preprocessing‐free approach		Yes	Yes	No	No	Yes	Yes	Yes
Chemical‐free process		Yes	Yes	No	Yes	Yes	Yes	Yes
Processing time [min]		30	30	15	<20	30–60	15–45	5
Application type		ICSI	ICSI	ICSI	ICSI	ICSI	IUI/ICSI	ICSI
Motility parameters	Factor of change between inlet and outlet	N/A	VSL ≈2.8 VCL ≈2.4	N/A	N/A	VSL = 1.3–3.8; VCL = 1.4–3.0	VSL = 1.2–1.4; VCL = 1.1–1.4	VSL = 1.6–5.0; VCL = 1.1–3.8
	% Motile	98%	N/A	N/A	N/A	≈95%	≈95%	≈99%
Biological parameters	Normal morphology	≈22%	≈27%	N/A	Q/R	N/A	30%	≈48%
	Nuclear maturity	N/A	N/A	N/A	N/A	N/A	≈40%	50%
	DNA fragmentation (DFI)	N/A	≈15%	N/A	<5%	N/A	<5%	4–6%
	Epigenetics aberrations	N/A	N/A	N/A	N/A	N/A	N/A	Yes
Model‐driven design		No	No	Yes	No	No	No	Yes

In the microfluidics realm, there are many surface designs decorating channels in various ways,^35^ including deterministic lateral displacement methods for separation of particles^36^, ^37^ and blood cells.^38^ To understand sperm motility in such complex geometries, many hydrodynamic models have been developed at different scales;^35^, ^36^, ^37^, ^38^, ^39^, ^40^, ^41^, ^42^, ^43^, ^44^, ^45^ however, to the best of our knowledge, no models exist that can bridge the necessary time and length scales to allow the modeling of clinically relevant microfluidic ARTs and improve their design parameters. Here, we designed a filter‐like microfluidic path using multiscale computer simulations in conjunction with experiments and examined how this artificial path of pillars interact with known descriptive and functional characteristics of human sperm, including sperm motility, morphology, DNA integrity, and methylome status. This multiscale model takes into account known hydrodynamic interactions between sperm cells and the complex boundaries, is scalable, and explains the rich and unexpected behavior of sperm cells as they traverse these anisotropic pillar geometries. The simulations predicted a unique range of periodicities that select for human sperm with best motility and morphology parameters. Using the predictions from the simulations as constraints, a novel microfluidic device—**S**imple **P**eriodic **AR**ray for **T**rapping **A**nd isolatio**N** (SPARTAN)—was built, and our experimental results confirmed model predictions about its performance. SPARTAN increased the persistence length of sperm, efficiently isolating highly motile, and morphologically normal sperm, with low epigenetic aberrance, from raw semen. Our approach provides a reliable and cost‐effective way to accurately and noninvasively select for sperm attributes relevant to a successful ART outcome.

## 2 Results and Discussion

### 2.1 Model‐Driven Design for Fabricating a Pillar Array Sorting Device

**Figure 1:**
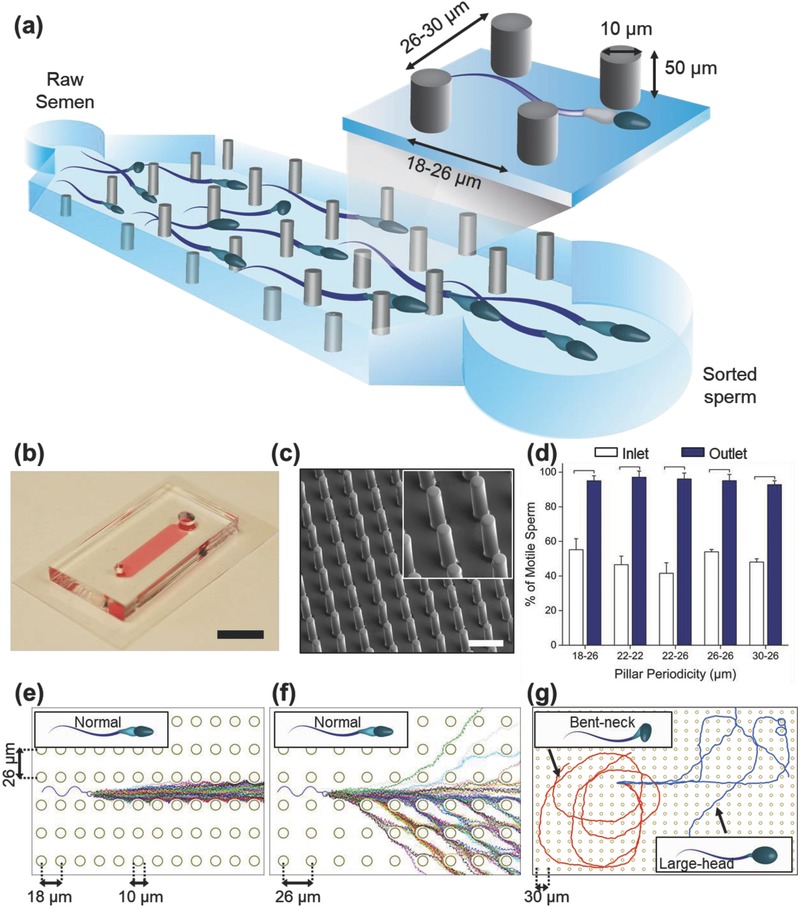
SPARTAN (**S**imple **P**eriodic **A**Rray for **T**rapping **A**nd isolatio**N**) for selecting motile and morphologically normal sperm. a) Schematic diagram of the microfluidic pillar array and an illustration of pillar dimensions. b) Photograph of the microfluidic device (scale bar: 10 mm). c) FESEM image of the 30 × 26 µm periodic pillar array (scale bar: 50 µm), and a close‐up FESEM image (inset) showing micropillars in detail (scale bar: 20 µm). d) Percentage of motile sperm at different pillar periodicities is plotted. Data are shown as average ± standard deviation. The brackets represent a statistically significant difference compared with the groups using one‐way ANOVA with Tukey's posthoc test for multiple comparisons (*N* = 3, *p* < 0.05). e,f) Simulated trajectories of (*n* = 100) normal morphology sperm in channels with 18 × 26 µm (left) and 26 × 26 µm (right) array periodicities. g) Simulated trajectories for sperm with abnormal morphology in a channel with 30 × 26 µm array periodicity. Trajectories of sperm with a bent neck (0.1π radians) and a larger head (×2 normal) are shown in red and blue, respectively.

SPARTAN (**Figure** **1**a–c) is designed using our multiscale hydrodynamic simulations (Experimental Section and Supporting Information) that model sperm swimming behavior in periodic pillar array geometries. In particular, the initial simulation results from the coarse‐grained multiparticle collision dynamics (MPCD) model revealed a wide variety of motility behavior for sperm traversing the pillar array, with the anisotropy of pillar spacing additionally influencing the swimming direction (Figure 1e,f; Figure S1, Supporting Information). Further, when pillars were spaced appropriately, sperm with morphological defects were effectively constrained due to their abnormal motility characteristics and were unable to traverse the channel effectively (Figure 1g). To quantitatively model this rather diverse and unexpected behavior at large time and length scales, we developed a multiscale model of sperm motility for the SPARTAN featuring microfluidic channels (Experimental Section and Supporting Information). Using these simulations, we calculated a range of candidate array geometries, ultimately choosing rectangular arrays with spacing values 18 × 26, 22 × 22, 22 × 26, 26 × 26, and 30 × 26 µm.

**Figure 2:**
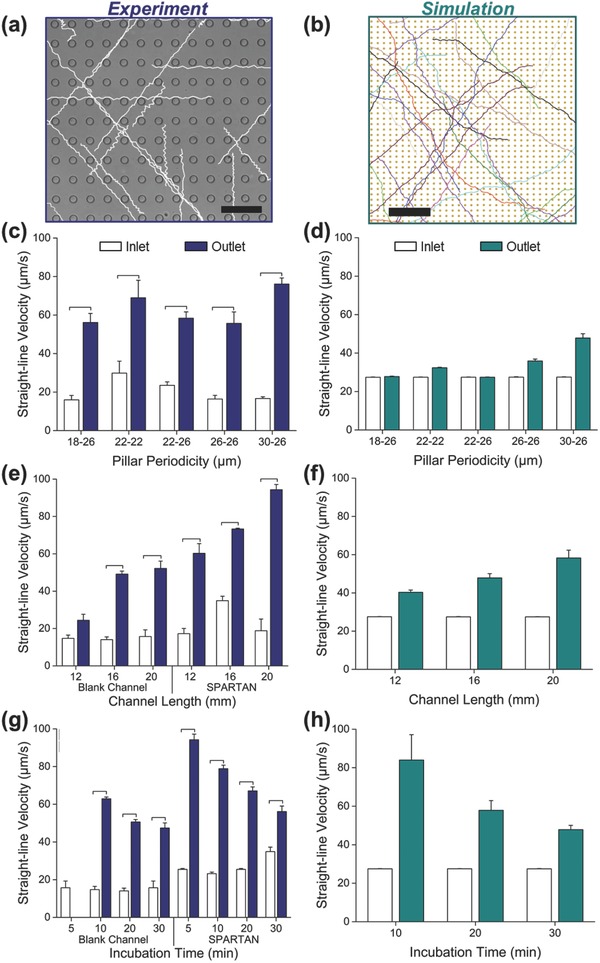
Optimization of SPARTAN for efficient sperm sorting. a) In vitro experimental sperm trajectories for the 30 × 26 µm pillar array compared with b) computer‐simulated sperm trajectories (multiscale model). Scale bars are 50 and 200 µm for experiments and simulations, respectively. Straight‐line velocity (VSL) values are compared at the inlet versus outlet after 30 min of incubation from c) experiments and d) simulations, for different pillar array periodicities. e) Experimental measurements of VSL values for channels with 30 × 26 µm array periodicity with varying lengths (30 min of incubation at outlet). f) VSL values obtained from the simulations after 30 min of incubation, for varying channel lengths with 30 × 26 µm array periodicity. g) Experimental measurements of VSL for channels with 30 × 26 µm array periodicity for varying incubation times, compared with the blank channel. h) VSL values obtained from the simulations for varying incubation times with 30 × 26 µm array periodicity. Data are shown as average ± standard deviation. The brackets represent a statistically significant difference compared with the groups using one‐way ANOVA with Tukey's posthoc test for multiple comparisons (*n* = 10–1700, *N* = 3, *p* < 0.05). *n*: number of sperm, *N*: number of experiments.

These predicted array spacings were then fabricated (Figure 1b,c), and the percentage of motile sperm at the outlet for all pillar periodicities were observed to be higher (>98%) than that of the raw semen at the device inlet (≈60%) (Figure 1d). To further evaluate the optimum spacing for SPARTAN, we identified sperm trajectories from both experiments and simulations. **Figure** **2**a,b illustrates trajectories for the 30 × 26 µm channels. At the inlet and outlet, sperm trajectories and kinematic parameters, including straight‐line velocity (VSL) and curvilinear velocity (VCL), were measured, and compared with blank microfluidic channels (BMC).^3^ As shown in Figure 2c, VSL values at the outlets of SPARTAN were higher than those at the inlet (Table S1, Supporting Information), consistent with the predictions from the multiscale model (Figure 2d). In particular, VSL (Figure 2c) and VCL (Figure S2a, Supporting Information) values and sperm recovery efficiencies (Figure S2b, Supporting Information) at the outlet of a 30–26 µm periodicity device were higher than that of all the other different periodicities considered. This enhanced motility and recovery efficiency of the 30–26 µm SPARTAN is a consequence of the hydrodynamic and boundary interactions between the sperm and pillar arrays, and is evident from the movies of tracked sperm shown in Videos S1–S5 in the Supporting Information.

### 2.2 Channel Length and Incubation Time Influences Sorting Efficiency

Using the multiscale model, we established the lengths from 12 to 20 mm, and incubation times from 5 to 30 min as candidate ranges for experiments with the 30–26 µm pillar periodicity SPARTAN. We then performed experiments to measure VSL and VCL at the inlet and outlet of these channels, and compared our results with BMC of similar length. As shown in Figure 2e, after 30 min of incubation, the sperm trajectory kinetic parameters VSL and VCL (Figure S3a, Supporting Information), measured at the outlets of the 20 mm length channels of both the SPARTAN and blank channels, were higher compared to the 12 and 16 mm long channels, with SPARTAN nearly doubling the observed velocities (Figure 2e; Table S2, Supporting Information). These results agree with the predictions of the simulations that at longer channel lengths, only highly motile sperm are able to reach the outlet, whereas sperm with low motility remain within the channel without reaching the outlet (Figure 2f).

**Figure 3:**
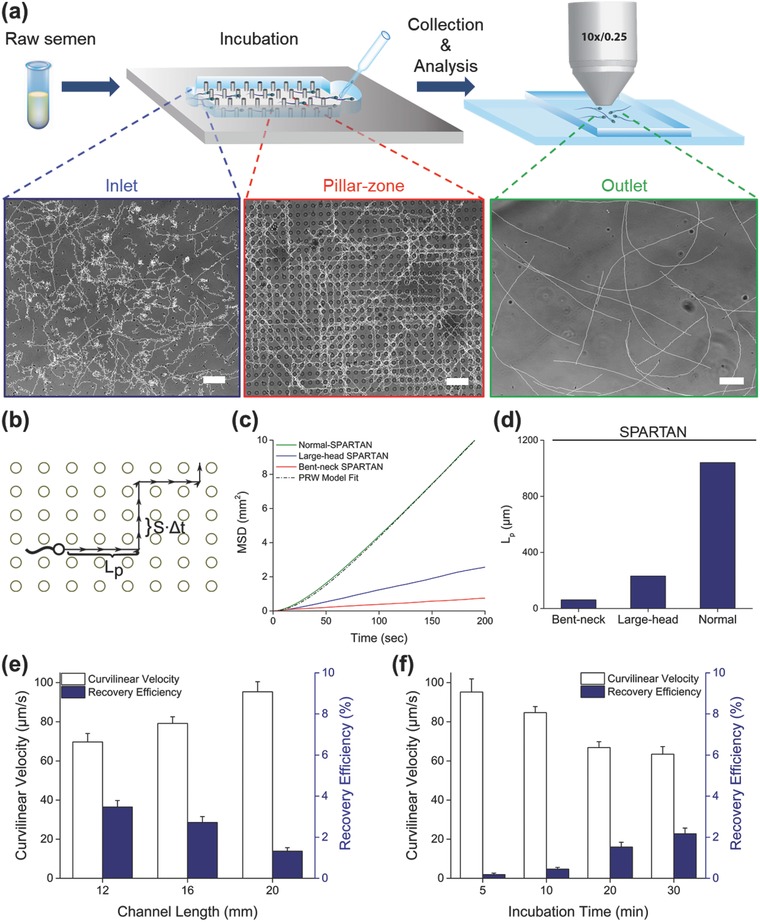
Analysis of sorted sperm motility, persistence, and recovery. a) Schematic illustrations of the sperm sorting process: semen is initially introduced into the device, allowed to incubate, and then output sperm are recovered and analyzed. Microscopy images of sperm trajectories traced using the CASA ImageJ plugin within the inlet, the pillar zone, and after collection from the outlet of the device are also shown (scale bars: 50 µm). b) Illustration of the persistent random walk (PRW) model parameters on a pillar array; *S* and *L*_p_ denote sperm velocity and persistence length, respectively. c) Mean‐squared‐displacement (MSD) values of normal and morphologically defective sperm calculated using the multiscale model. d) Analysis of the persistence length, *L*_p_, values for normal and morphologically defective sperm calculated using the multiscale model. e) Experimental measurements of VCL for sperm and recovery efficiency at the outlet of SPARTAN for different channel lengths. f) Experimental measurements of VCL for sperm and recovery efficiency at the outlet of SPARTAN for various incubation times. Experimental data are shown as average ± standard deviation (*n* = 10–1600, *N* = 3). *n*: number of sperm, *N*: number of experiments.

Since sperm recovery rate increases with decreased channel length (**Figure** **3**e), incubation time experiments were conducted using 16 mm long devices, corresponding to the middle of the range shown in Figure 2e,f. As shown in Figure 2g and Figure S3b (Supporting Information), VSL and VCL values were higher when incubation time was decreased from 30 to 10 min, with the highest sperm motility values observed at the outlet within 5 min of incubation in SPARTAN, in agreement with the model predictions (Figure 2h). In contrast, no sperm were observed at blank channel outlets after the same incubation time (Table S3, Supporting Information). The resulting VSL and VCL distributions for sorted sperm at the outlet of SPARTAN channels are shown in Figures S4 and S5 (Supporting Information) for different incubation times.

### 2.3 Periodic Pillar Arrays Enhance Sperm Persistence Length

As shown in Figure 3a (Videos S1–S5, Supporting Information), sperm trajectories in the pillar array persist longer in a straight path compared to sperm trajectories outside the array, such as at both the inlet and the outlet. This observation can be quantitatively described by two parameters as illustrated in Figure 3b. Here, *S* corresponds to the average velocity of a single sperm, and *L*_p_ denotes the persistence length—the distance a sperm moves on average before changing direction. To evaluate the effect of this persistence length enhancement on sperm recovery efficiency, we used our multiscale model of sperm (Experimental Section and Supporting Information) that allows us to simulate large sperm populations over the course of 30 min of incubation time. We quantified these trajectories using mean‐squared‐displacement (MSD) analysis (Experimental Section) and fit them to the persistent‐random‐walk (PRW) model (Figure 3c).^39^

MSD measurements of normal and morphologically defective sperm obtained using the multiscale model indicated that normal morphology sperm in SPARTAN demonstrate the largest increase in MSD values. Further analysis using the PRW model reveals a large enhancement of the distance traversed by the sperm in SPARTAN, compared to the BMC.^3^ More specifically, the persistence length, *L*_p_, for normal sperm is 5‐ and 20‐fold larger than that of sperm with large head and bent neck, respectively (Figure 3d). The simulated trajectories shown in Video S6 (Supporting Information) differ for sperm with normal and defective morphologies, as they move through the SPARTAN channel. This increase in the persistence length of sperm with normal morphology is due to boundary and hydrodynamic interactions within the pillar array, and is the principle behind the successful sorting of sperm using SPARTAN as it increases the selective separation between the morphologically normal and defective sperm.

### 2.4 Sperm Recovery Efficiency Increases with Shorter Device Length and Longer Incubation

To evaluate the effect of channel length and incubation time on sperm recovery efficiency, we analyzed the number of sperm in the inlet, pillar, and the outlet zones (Figure 3a). The measured VCL values and recovery rates at the outlet are shown in Figure 3e,f, for varying channel lengths and incubation times. As shown in Figure 3e, sperm recovery efficiency increased with decreasing channel length, having its highest value after 30 min of incubation. As expected, increased incubation time allows more sperm to arrive at the outlet, while lowering the observed velocity values (Figure 3f).

### 2.5 SPARTAN Improves Sperm Morphology by Twofold

**Figure 4:**
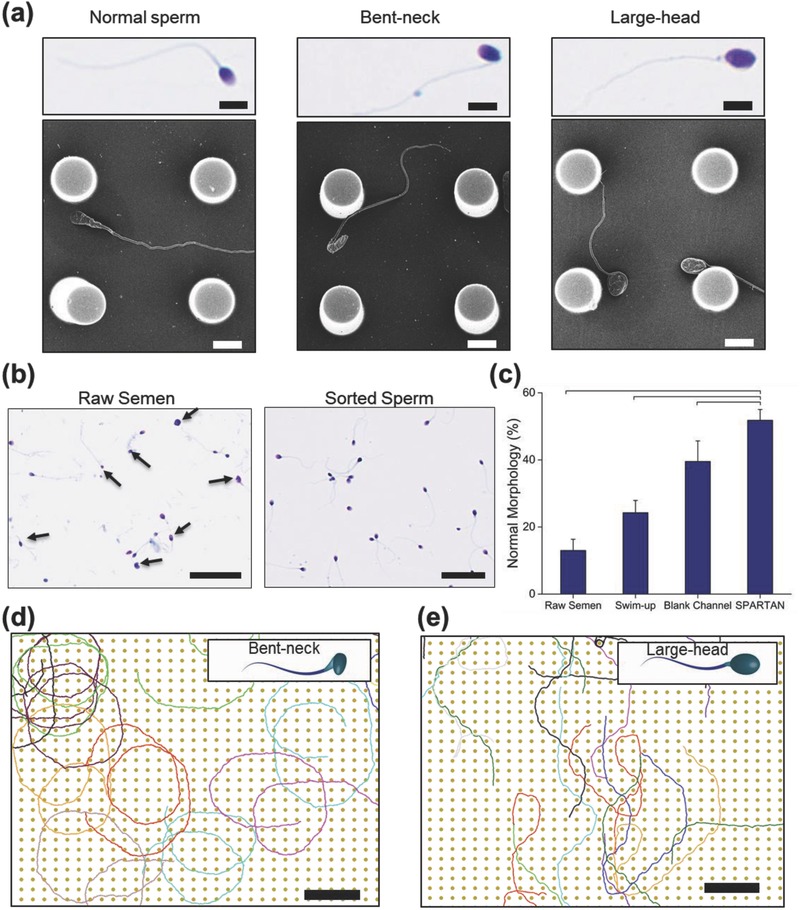
Sperm morphological analysis. a) Microscopy images of Quick III‐stained sperm together with corresponding FESEM images illustrating several morphological defects: (i) normal, (ii) bent‐neck, and (iii) large‐head (scale bars: 10 µm). b) Microscopy images of Quick III‐stained raw semen (arrows show abnormal sperm) and sperm sorted using SPARTAN (scale bars: 50 µm). c) Morphology analysis (based Kruger's strict criteria) of raw semen, sperm processed through the swim‐up technique, the blank microfluidic channel (BMC), and SPARTAN. Data are shown as average ± standard deviation. The brackets represent a statistically significant difference compared with the groups using one‐way ANOVA with Tukey's posthoc test for multiple comparisons (*n* = 200; *N* = 3; *p* < 0.05). *n*: number of sperm, *N*: number of experiments. d,e) Sperm trajectories from the coarse‐grained MPCD simulations for the 30 × 26 µm pillar array periodicity: d) Sperm with a bent neck (0.1 π radians) make circles, preventing themselves from leaving the pillar array (scale bars: 200 µm); e) Sperm with a three times larger head diameter than normal get stuck and make sudden turns.

Here, and in the following sections, biological characteristics of sperm sorted via SPARTAN were compared with those sorted by the BMC, the swim‐up technique, and those from raw semen. Representative stained and field emission scanning electron microscope (FESEM) images (Experimental Section) of morphologically defective sperm, raw semen, and sperm sorted using SPARTAN are shown in **Figure** **4**a–c. As shown in Figure 4c, there is a higher percentage of morphologically normal sperm (≈52%) sorted by SPARTAN compared to sperm sorted using BMC (≈40%), the swim‐up technique (≈24%), and sperm from raw semen (13%). Videos S7 and S8 (Supporting Information) illustrate that sperm with morphological defects can get stuck within the pillar array, and thus do not reach the outlet. Various morphological defects observed in the experiments are shown in Figure S6 in the Supporting Information. For instance, our simulation results show that even motile sperm with bent neck (Figure 4d) or large head (Figure 4e) cannot travel in straight lines in the pillar array, resulting in reduced forward motility, i.e., a lower sperm recovery rate at the outlet (Videos S9–S11, Supporting Information).

### 2.6 Sperm DNA Integrity is Enhanced in SPARTAN‐Sorted Sperm

**Figure 5:**
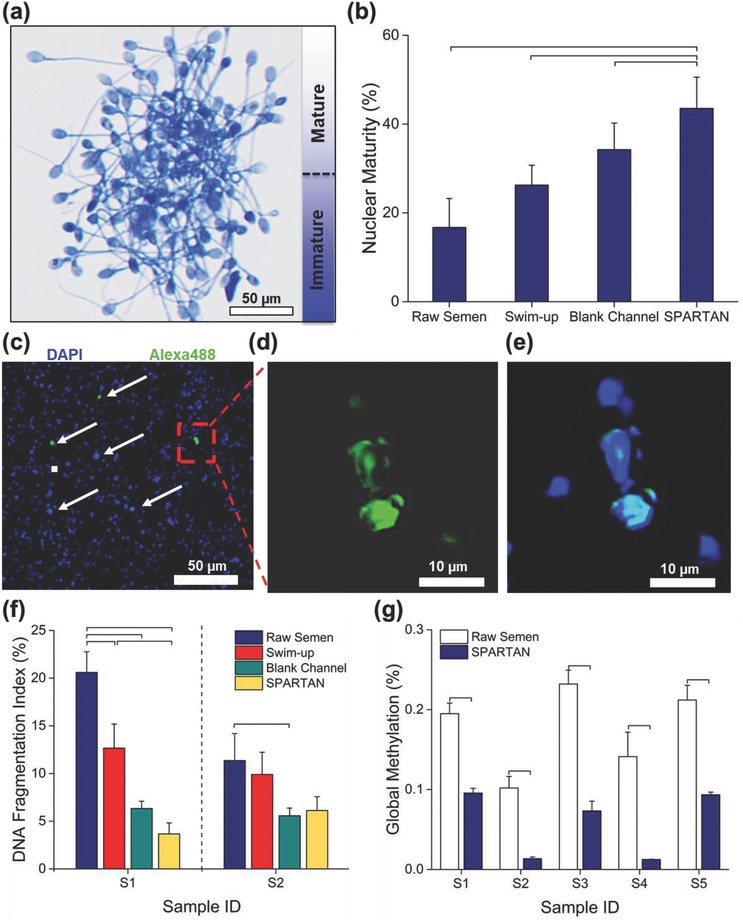
Analysis of sperm DNA integrity. a) Microscopy image of acidic aniline blue staining shows different stages of nuclear maturity of sperm. b) Nuclear maturity percentage of sperm sorted using SPARTAN compared with sperm processed by the swim‐up technique, and using the blank microfluidic channel (BMC). Comparison to raw semen sample is also shown (*n* = 5 semen samples). c–e) Fluorescence staining images of DNA fragmentation analysis using TUNNEL assay in raw semen, which is stained with Alexa 488 (green fluorescent), counter stained with 4′,6 diamidino‐2‐phylindole (DAPI) (blue fluorescent). f) Analysis of DNA fragmentation index (DFI) of two sperm samples (S1 and S2) sorted by SPARTAN compared with sperm processed by swim‐up technique and blank microfluidic channel (BMC). Comparison to raw semen sample is also shown (*n* = 44–353, *N* = 3). g) Sperm DNA global methylation level analysis of raw semen and sperm sorted by SPARTAN. Data are shown as average ± standard deviation. The brackets represent a statistically significant difference compared with the groups using one‐way ANOVA with Tukey's posthoc test for multiple comparisons (*n* = 200; *N* = 3; *p* < 0.05). *n*: number of sperm, *N*: number of experiments.

Evaluation of chromatin condensation in sorted sperm (**Figure** **5**a, Experimental Section), revealed a higher percentage of mature sperm (43.5 ± 7.1%) using SPARTAN, nearly a threefold increase compared to raw semen, and also, approximately a twofold increase compared to swim‐up technique frequently used in the clinics (Figure 5b). We then evaluated DNA fragmentation of two samples (Sample 1: S1 and Sample 2: S2) sorted by the swim‐up technique, BMC, and SPARTAN. Since each sample's inherent parameters such as DNA fragmentation is unique, we observed higher DNA fragmentation index (DFI, Experimental Section) in S1 whereas DFI was lower in S2. In sample S1, we observed that the DFI of sperm sorted by SPARTAN was more than a six‐fold lower than that of raw semen samples; nearly a fourfold lower than that of swim‐up technique, and also, approximately a twofold lower than that of BMC (Figure 5c–f). In sample S2, we observed that the DFI of sperm sorted by SPARTAN was nearly twofold lower than that of raw semen samples and swim‐up technique. In addition, as shown in Figure 5g, the relative global methylation level (Experimental Section) was higher in raw semen samples than that in sperm sorted using SPARTAN. All data on morphology and nuclear maturity for sperm sorted via SPARTAN are shown in Table S4 (Supporting Information), in comparison to raw semen data, swim‐up technique, and blank microfluidic channel (BMC) data.

## 3 Conclusion

The sperm journey through the female reproductive tract—conserved in viviparous mammals throughout millions of years of evolution—is a naturally effective “filter” for fertile sperm. Inspired by this, SPARTAN—designed using an experimentally validated model‐driven approach—not only acts as a filter, but, as demonstrated by our modeling results, also increases the directional persistence of sperm within its pillar array, further separating populations of progressive and nonprogressive sperm. While one might expect any swimmer in a constrained environment to behave more persistently, we find that sperm with large, amorphous heads, or bent necks have entirely different trajectories than morphologically normal sperm in SPARTAN.

Moreover, SPARTAN allows sperm to rapidly self‐sort without the need for pumps, chemoattractants, or flow, and eliminates lengthy centrifugation or washing steps that are shown to lead to ROS formation (see Table 1).^6^ It offers a simple, clinically applicable one‐step procedure to successfully retrieve highly motile, and morphologically normal human sperm with high DNA integrity. In addition, SPARTAN's ability to sort sperm for low global epigenetic aberrations is unprecedented, and has the potential to improve quality of sperm selected for ICSI. In comparison to other methods of sperm preparation, such as swim‐up, and the BMC developed earlier in our laboratory, SPARTAN sorting of sperm significantly reduced assay time (swim‐up: 60–90 min, BMC: 30–45 min, and SPARTAN: 10 min), reduced DNA fragmentation (swim‐up: ≈13%; BMC: ≈6%; and SPARTAN: 4–6%), improved morphology (swim‐up: ≈24%, BMC: ≈40%, SPARTAN: ≈52%), and nuclear maturity (swim‐up: ≈26%, BMC: ≈34%, and SPARTAN: ≈44%).

It is important to note that in the current form of the device, the sperm recovery rate makes SPARTAN more appropriate for ICSI,^40^ which uses a single sperm, than techniques that need a larger number of sperm cells, such as intrauterine insemination (IUI).^41^ As we have shown earlier,^6^ device recovery performance can potentially be extended beyond ICSI applications, and sperm yield can be increased by integrating a large reservoir to collect higher cell numbers and make the device more appropriate for IUI applications.

For over 30 years, it has been generally accepted in the field that sperm morphology has some relationship to its fertility potential. The studied correlation of sperm morphology with fertility links abnormal sperm shape to reduced oocyte fertilization for in vitro fertilization.^42^, ^43^ However, other correlations, such as the relationship between the shape of sperm and natural conception rates, have been much harder to demonstrate.^44^, ^45^ This microfluidic study offers new evidence that sperm morphology is indeed a significant factor in sperm transport in vitro. In addition, our results have implications beyond human fertility including biodiversity and conservation of endangered or rare species. Moreover, the similarities in fluid physics of swimmers across different species,^46^ from bacteria to parasites, make these findings broadly applicable to other motile microorganisms. In fact, many such active swimmers across diverse biological systems have been an inspiration for the design of artificial micrometer‐scale flagellar systems including biohybrid microrobots,^47^, ^48^, ^49^, ^50^, ^51^, ^52^ and we also envision broad applications of our findings in areas such as medical diagnosis,^53^ biosensing,^54^ and targeted drug delivery.^55^

## 4 Experimental Section

*Sample Collection and Preparation*: Sperm sorting analysis was performed using deidentified discarded human semen samples from Reproductive Endocrinology and Infertility Laboratory, Fertility and Reproductive Health Services, Stanford University School of Medicine, Stanford University. All sperm processing experiments were performed within 1–3 h after the semen samples were collected from the clinical laboratory. An exempt human subjects protocol (ID:30538) was approved by Stanford IRB committee.

*Swim‐Up Method*: Raw semen was diluted with 9 mL of human tubal fluid (HTF) containing 4% bovine serum albumin (BSA). The diluted sperm suspension was then centrifuged at 500 × *g* for 5 min. The supernatant was removed and discarded. The remaining pellet was washed again in HTF/BSA and centrifuged at 500 × *g* for 5 min. The supernatant was again discarded. Finally, 500 µL of similar media was added slowly along the sidewall of the centrifuge tube to avoid pellet disruption. The sample was then placed in a 37 °C incubator, and motile sperm were allowed to swim up, out of pellet, for 30 min. The motile sperm were collected within the media and used for further study.

*Device Fabrication*: SPARTAN features high aspect ratio pillar arrays. The overall dimensions of the fabricated microfluidic devices had lengths ranging from 14 to 22 mm (with pillar array lengths in the range 12–20 mm), a width of 4 mm, and a channel height of 50 µm. The diameters of the inlet and outlet chambers were 1.2 and 3 mm, respectively. In the middle of the channel, 10 µm diameter pillars were placed with various spacing values based on simulation results. A brief schematic of the SPARTAN device is shown in Figure 1a. The device was fabricated using standard soft lithography. In brief, a 50 µm thick layer of SU‐8 photoresist was coated and developed on a 4 in. silicon wafer, creating a microchannel template. Subsequently, the SPARTAN featuring devices were fabricated using Sylgard 184 (Dow Corning, Midland, MI, USA) in a 10:1 v/v ratio of base versus curing agent that was poured onto the silicon wafer, degassed, and cured at 70 °C for 2 h. After curing, inlet and outlet chambers were punched using Acu Punch (tips 1.0 and 2.0 mm). The resulting channel was sealed on a glass slide using oxygen plasma and baked at 80 °C for 30 min before use (Figure 1b). An FESEM image of the unpopulated periodic pillar array is shown in Figure 1c.

*FESEM Sample Preparation and Imaging*: After 10 min of incubation, the sperm sorting process was stopped, and the device was left for air‐drying to attach sperm cells on the surface. After air‐drying, sperm were fixed on the SPARTAN featuring device using 2% glutaraldehyde with 4% paraformaldehyde in 0.1 m Na Cacodylate buffer (pH 7.3) at 4 °C for 4 h; the chip was then treated with 1% osmium tetroxide for 1 h at 4 °C. After washing with mQ‐H_2_O, sperm was dehydrated with 50%, 70%, 80%, and 95% ethanol (200 proof) solutions prepared in water and pure ethanol for 15 min. The sample was dried using a critical‐point dryer (Tousimis Autosamdri‐815) and then coated with copper using a sputtering system (Cressington 208 HR). Prepared samples were imaged using a Zeiss Sigma FESEM at Beckmann center at Stanford University.

*Microfluidic Sperm Sorting and Analysis*: The microfluidic device was first filled with fresh sperm washing media, and after which raw semen sample was added to the channel inlet. A thin layer of sterile embryo‐tested mineral oil was placed on top of the media in the inlet and outlet to avoid evaporation. The microfluidic device was then placed into the incubator at 37 °C for various incubation times as shown in Figure 2. After incubation, the sperm inside the channel as well as at the inlets and outlets were imaged using bright field microscopy (Zeiss, Germany), and videos were recorded at multiple locations for a duration of 10 s at a rate of 10 frames per second (10×). These videos were then converted to image sequences using ZEN lite Image Analysis Software. Following this step, these images were uploaded into ImageJ (National Institute of Health), and the ImageJ CASA plugin^56^ was used to measure motility parameters, such as VSL and VCL (Figure S7, Supporting Information). In addition to motility parameters, percentage of motile sperm and recovery rates were also calculated. The percentage of motile sperm was defined as the fraction of motile sperm relative to the total sperm count. Recovery percentage was calculated based on the total number of sperm collected in channel outlet at various incubation times (5, 10, 20, and 30 min) relative to the total number of sperm in raw semen injected at channel inlet. The percentage of collectable sperm was calculated by dividing the total sperm count collected from the outlet channel by the total sperm count introduced into the microchannel.

*Multiscale Modeling of Sperm Motility*: To be able to simulate large ensembles of sperm cells and study their motility over longer time scales in realistic device geometries, a probabilistic lattice model was built which was trained via coarse‐grained hydrodynamic simulations (MPCD, see the following) (Figure S8, Supporting Information). This multiscale approach was allowed to simulate the motion of the large ensembles (>>1000) sperm in pillar geometries over the course of minutes to hours, with full effects of hydrodynamics. Detailed explanation of this approach for single sperm cell motility is given in ref. 57, but here a brief description is provided that explains how it is generalized to a large population of sperm cells (with different morphologies) with a given velocity distribution, in realistic device geometries.

For the coarse‐grained simulations, a well‐established particle‐based simulation technique called MPCD—also known as stochastic rotation dynamics (SRD)—was used.^58^, ^59^, ^60^ This technique uses a large number of coarse‐grained solvent particles to efficiently model the behavior of a fluid, including embedded structures and active swimmers. Transport behavior of MPCD was well characterized by this study and others,^59^, ^60^, ^61^, ^62^, ^63^, ^64^, ^65^ and applied to a diverse set of problems.^66^, ^67^, ^68^, ^69^ including sperm motility and cooperation.^46^, ^70^, ^71^ In the MPCD simulations, the solvent dynamics was consisted of a streaming and a collision step, during which particles exchange momentum within a collision cell of linear dimension *a* (Figure S8, Supporting Information).^58^, ^62^, ^63^ This simulation region was consisted of a box of dimensions that are six times larger than the postspacing in a given linear dimension; distances in the simulations were measured in units of this cell size. Average density of solvent particles was chosen to be 10 in these units, with solvent mass set to unity. A simulation time step of τ = 0.025 and a collision angle of 90° were used.^57^

The sperm was modeled as a semiflexible (bending rigidity of 3000 *k*_B_ *T*, where *k*_B_ is the Boltzmann constant and *T* is the temperature) bead‐spring polymer of length about 60 (in units of collision cell size a), with stiff springs as described in the literature.^57^, ^71^ The pillars were modeled as series of immobile beads with a heavier mass (×5 of solvent particle mass), and the Lennard–Jones repulsion of Yang et al.^71^ was used for the forces between the walls/pillars and the sperm (Figure S8, Supporting Information). The beads of the bead‐spring polymer (with ×10 solvent particle mass) participated in the MPCD collisions along with regular fluid particles, thus exchanging momentum and providing the fluid–solid coupling.^72^ A velocity‐Verlet scheme was used to perform the molecular dynamics step for the sperm backbone using a time step of τ/100. Finally, a traveling wave of the form *c*(*i*, *t*) = sin(*ki* + *ωt*) was imposed on the angle of the *i*th bond of the sperm tail, to mimic sperm shape and swimming motion, with *k* = 0.2 and ω = 0.0005 π/τ.^57^, ^70^, ^71^ Finally, the power input into the system due to swimming of sperm was balanced using a thermostat.^71^ Further implementation details, including an efficient GPU (Graphics Processing Unit) implementation of the solvent algorithm,^73^ can be found in ref. 57.

The MPCD model produced microscopic trajectories (*n* = 100, see Figure S1, for example, in the Supporting Information) which were then used to train a probabilistic rule‐based lattice model to describe the sperm's movement in any given pillar geometry for realistic time and length scales. This probabilistic model considers that there are a finite number of directions a sperm could face in a post lattice (*m* = 11–13 in this case),^57^ and that the behavior of its movement will depend on what direction it faces. This was done by specifying, for each directional state, probabilities of transitioning to the previous or next state, as well as probabilities of moving in cardinal directions along the lattice. Specifically, a probability of rotation was defined from direction *d* to direction *e* as *P*_r_(*e*,*d*), as well as a probability of moving (or staying) along the lattice in a cardinal direction (ζ = stay, left, up, right, down), given the current direction *d* as *P*_m_(ζ,*d*). These rules were alternated for the amount of time desired for the simulation and were used to rapidly simulate a large population of sperm in a given pillar array channel. Figure S9 (Supporting Information) illustrates the use of these probabilities. Once again, the reader is referred to ref. 57 for further algorithmic details.

Figure S10 (Supporting Information) shows a flowchart of this multiscale approach, and all the probability rules obtained using the MPCD simulations for different configurations and sperm morphologies are given in Tables S5–S7 (Supporting Information). Experimentally measured inlet velocity distributions (Figure S4b, Supporting Information) were then used as input to the model to produce the simulation results given in Figure 2. This was done by sampling from the distribution (Figure S4, Supporting Information) to get the velocities of each sperm in the lattice model run and converting the performance of the MPCD‐based sperm (in units of *a* and τ) into micrometer and *T*, resulting in a number of time steps to simulate. Here, *T* corresponded to one tail beat cycle for the sperm.

The lattice simulations were initialized with the sperm distributed across the entrance of the periodic array, with sperm heads facing the array entrance. While this was an artificial initial condition, the effects of all such transient distributions vanished relatively quickly, as shown in Video S6 (Supporting Information), since sperm rapidly approach equilibrium and randomize their orientations as they move along the channels.

Using this multiscale model, SPARTAN was optimized to produce the maximum increase in VCL between the sperm observed in the output chamber and those introduced in the input, as defined by the ratio VSL_out_/VSL_in_.

*PRW Model*: Besides the description via velocities (VCL and VSL), the sperm trajectories can also be analyzed by calculating the MSD. Assuming that the motion of the sperm is restricted to two dimensions, the MSD is given by (Equation (1))

(1) $$\left\langle d^{2}\left(t\right)\right\rangle =\left\langle {\left(x\left(t\right)-x\left(0\right)\right)}^{2}\right\rangle +\left\langle {\left(y\left(t\right)-y\left(0\right)\right)}^{2}\right\rangle$$

It was found that the motion of sperm was ballistic at short times $\left\langle d^{2}\left(t\right)\right\rangle \sim t^{2}$, and diffusive at long times $\left\langle d^{2}\left(t\right)\right\rangle \sim t$. This type of behavior can be described by the PRW model.^39^ In this model, the MSD took the form (Equation (2))

(2) $$\left\langle d^{2}\left(t\right)\right\rangle =2L_{p}\left[St-L_{p}\left(1-e^{St/L_{p}}\right)\right]$$

where *S* denotes the velocity of the sperm and *L*_p_ corresponds to the persistence length of the sperm. This approach can successfully describe the short time, i.e., $\left\langle d^{2}\left(t\right)\right\rangle \approx S^{2}t^{2}$ for *t* << *L*_p_/*S*, and the long time, $\left\langle d^{2}\left(t\right)\right\rangle \approx 2SL_{p}t$ for *t* >> *L*_p_/*S*, behavior of sperm trajectories.^3^

*Sperm Morphology Analysis*: Sperm were collected from the outlet and evaluated morphologically using Quick III sperm morphology kit.^74^ Briefly, sperm were collected at the channel outlet 30 min after loading into the inlet, and smeared on the glass slide. After air‐drying, glass slides were incubated in staining buffers including fixative, buffers number 1 and 2 sequentially at 1 min interval. After drying, glass slides were imaged using a NanoZoomer 2.0 RS with 40× magnification (Hamamatsu). Based on the generated images, sperm were classified into different morphologies as described previously by Bartoov et al.^75^

*Chromatin Condensation Analysis*: Sperm sorted using SPARTAN were subjected to chromatin condensation analysis using the aniline dye blue test (a dye that selectively stains lysine‐rich histone proteins) and compared with sperm sorted by swim‐up and unprocessed semen as previously described.^17^ Briefly, sperm were washed twice in phosphate‐buffered saline (PBS), treated with 1% sodium dodecyl sulfate in sodium citrate, spread onto a glass slide and allowed to dry. Followed by fixation in 3% buffered glutaraldehyde in 0.2 m PBS for 20 min, slides were stained with 5% aqueous aniline blue mixed with 4% acetic acid for 15 min at room temperature. Finally, 200 sperm cells were evaluated using NanoZoomer 2.0 RS with 40× magnification (Hamamatsu). The percentage of dark blue stained sperm heads (an indicator of abnormal chromatin decondensation) was calculated. Samples with more than 30% of sperm showing dark blue staining were considered abnormal.

*Sperm DNA Fragmentation Analysis*: DNA fragmentation was performed with sperm sorted using SPARTAN, the swim‐up technique, and sperm from raw semen. The sperm collected from the outlet were spread out over the silanized glass slide and fixed with methanol/acetic acid mixture. Cells were permeabilized with PBS containing 1% Triton ×100. Cells with fragmented DNA were revealed using an APO‐BrdU Terminal deoxynucleotidyl transferase (TdT) dUTP (deoxyuridine triphosphate) Nick‐End Labeling (TUNEL) assay kit (Thermo Fisher Scientific). Sperm with fragmented DNA were detected using DNA labeling buffer containing TdT Enzyme and BrdU, which specifically binds to nicked DNA. Finally, fluorescently labeled anti‐BrdU antibody was used to identify sperm with fragmented DNA nuclear color (Alexa 488), whereas other cells have blue nuclei (4′,6 diamidino‐2‐phylindole (DAPI), counter stain). On each slide, ≈200 sperm cells were counted, and a DFI was calculated.

*Sperm Global Methylation Analysis*: Global DNA methylation analysis was conducted using an enzyme‐linked immunosarbent assay (ELISA)‐based Methyl‐Flash Methylated DNA Quantification kit (Epigentek Group Inc., Farmingdale, NY, USA).^76^ Briefly, 100 ng of genomic DNA was isolated from sperm sorted using SPARTAN and raw semen. A standard curve was generated using negative and positive internal controls in the same plate, and the amount of methylated DNA was calculated according to the formula provided in the manufacturer's protocol.

*Statistical Methods*: To evaluate the significance of various measurements reported, one‐way analysis of variance (ANOVA) with Tukey's posthoc test was performed for multiple comparisons with statistical significance threshold set at 0.05 (*p* < 0.05). Data were presented as average ± standard deviation. Sample size was indicated at each figure caption.

## Conflict of Interest

Utkan Demirci (U.D.) is a founder of and has an equity interest in the following: (i) DxNow Inc., a company that is developing microfluidic and imaging technologies for point‐of‐care diagnostic solutions; (ii) Koek Biotech, a company that is developing microfluidic IVF technologies for clinical solutions; and (iii) Levitas Inc., a company focusing on developing products for liquid biopsy. U.D.'s interests were viewed and managed in accordance with the conflict of interest policies.

## Supporting information

Supplementary

Supplementary

Supplementary

Supplementary

Supplementary

Supplementary

Supplementary

Supplementary

Supplementary

Supplementary

Supplementary

Supplementary
