# Supplementary material for: Guidance and Self‐Sorting of Active Swimmers: 3D Periodic Arrays Increase Persistence Length of Human Sperm Selecting for the Fittest
Source: Adv Sci (Weinh). 2017 Dec 27;5(2):1700531. doi: 10.1002/advs.201700531 (PMC5827459; doi:10.1002/advs.201700531)
Supplement: Supplementary file 1 — Supplementary [file ADVS-5-1700531-s002.pdf]

Copyright WILEY-VCH Verlag GmbH & Co. KGaA, 69469 Weinheim, Germany, 2017.

ADVANCED SCIENCE
Open Access

Supporting Information

for *Adv. Sci.*, DOI: 10.1002/advs. 201700531

Guidance and Self-Sorting of Active Swimmers: 3D Periodic Arrays Increase Persistence Length of Human Sperm Selecting for the Fittest

Thiruppathiraja Chinnasamy, James L. Kingsley, Fatih Inci, Paul J. Turek, Mitchell P. Rosen, Barry Behr, Erkan Tüzel, and Utkan Demirci**

Copyright WILEY-VCH Verlag GmbH & Co. KGaA, 69469 Weinheim, Germany, 2016.

Supporting Information

Guidance and self-sorting of active swimmers: 3-D periodic arrays increase persistence length of human sperm selecting for the fittest

Thiruppathiraja Chinnasamy[*], *James L. Kingsley*[*], *Fatih Inci*, *Paul J. Turek*, *Mitchell P. Rosen*, *Barry Behr*, *Erkan Tüzel*[**], *Utkan Demirci*[**].

Dr. Thiruppathiraja Chinnasamy, Dr. Fatih Inci, Prof. Utkan Demirci
Bio-Acoustic MEMS in Medicine (BAMM) Laboratory
Canary Center at Stanford for Cancer Early Detection
Department of Radiology, Stanford School of Medicine, Stanford University, Palo Alto, CA, USA

Mr. James L. Kingsley, Prof. Erkan Tüzel
Department of Physics, Worcester Polytechnic Institute, Worcester, MA, USA

Dr. Paul J. Turek
The Turek Clinic, San Francisco, CA, USA

Dr. Mitchell P. Rosen
Department of OBGYN, University of California San Francisco School of Medicine, San Francisco, CA, USA

Prof. Barry Behr
Department of Obstetrics and Gynecology, School of Medicine, Stanford University, Stanford, CA, USA

* co-first authors: Thiruppathiraja Chinnasamy, Ph.D., and James L. Kingsley, Ph.D.
** co-corresponding authors: Utkan Demirci, Ph.D. (email: utkan@stanford.edu) and Erkan Tüzel, Ph.D. (email: etuzel@wpi.edumailto:etuzel@mailaps.org)

Supplementary Figures

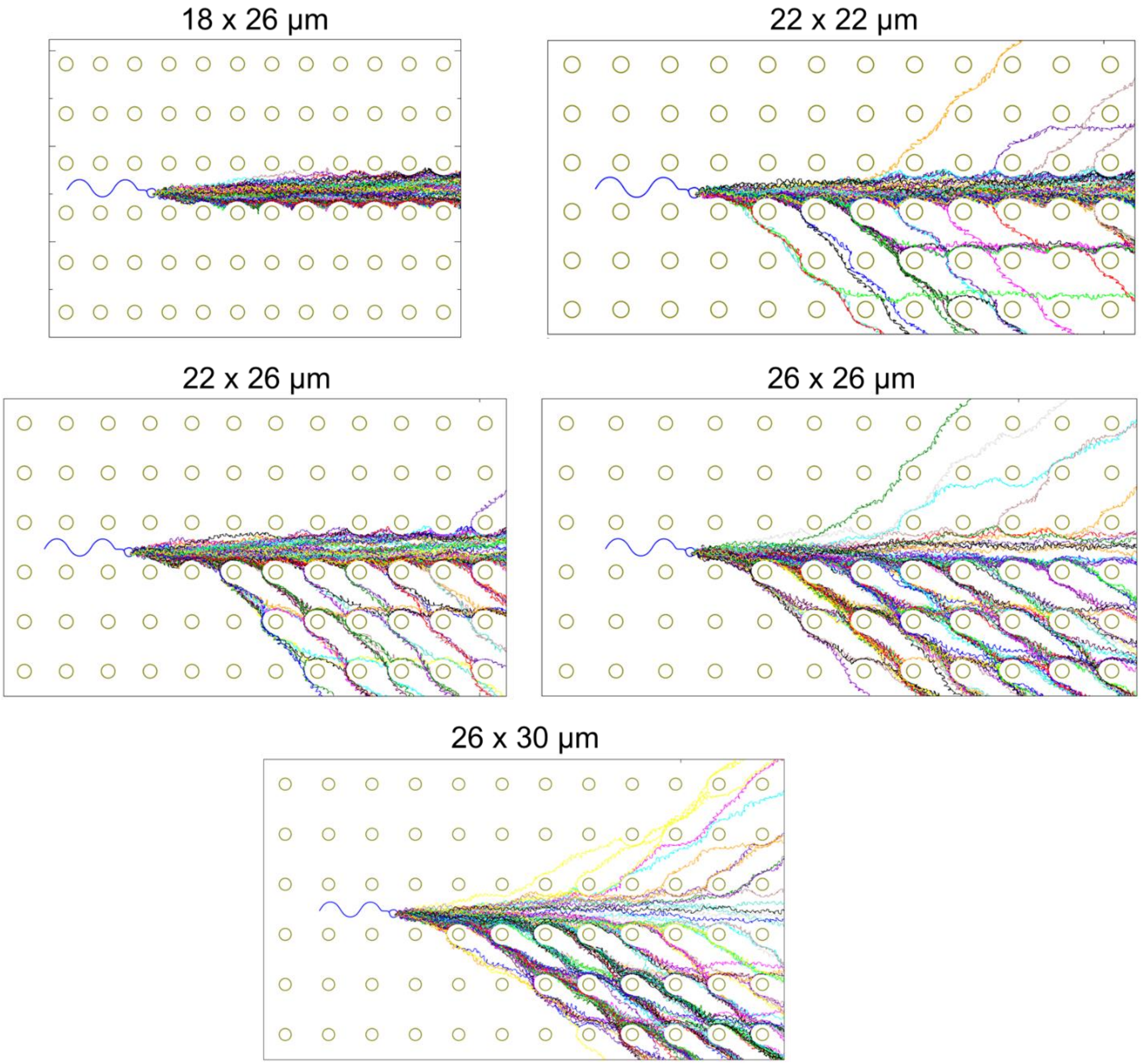

Figure S1. Simulated trajectories of (n=100) normal morphology sperm in channels with various array periodicities.

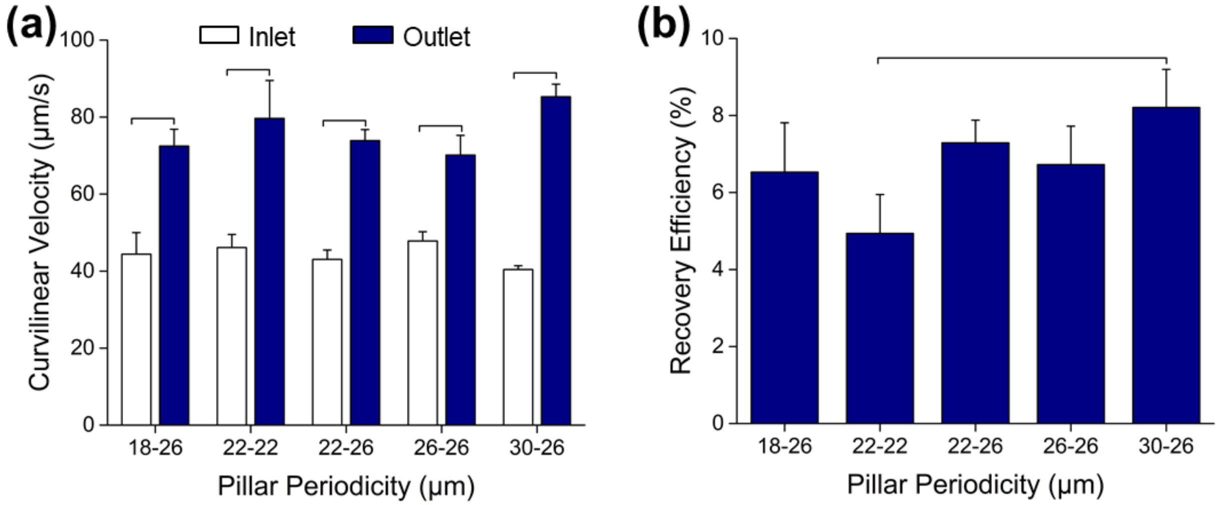

Figure S2: Analysis of collectable curvilinear velocity (VCL) and sperm recovery. (a) VCL data calculated at the inlet and outlet for different pillar array periodicities. (b) Recovery efficiency for motile sperm from different pillar array periodicity devices. All data was collected on the channels with 12 mm length after 30 minutes of incubation time at the outlet.

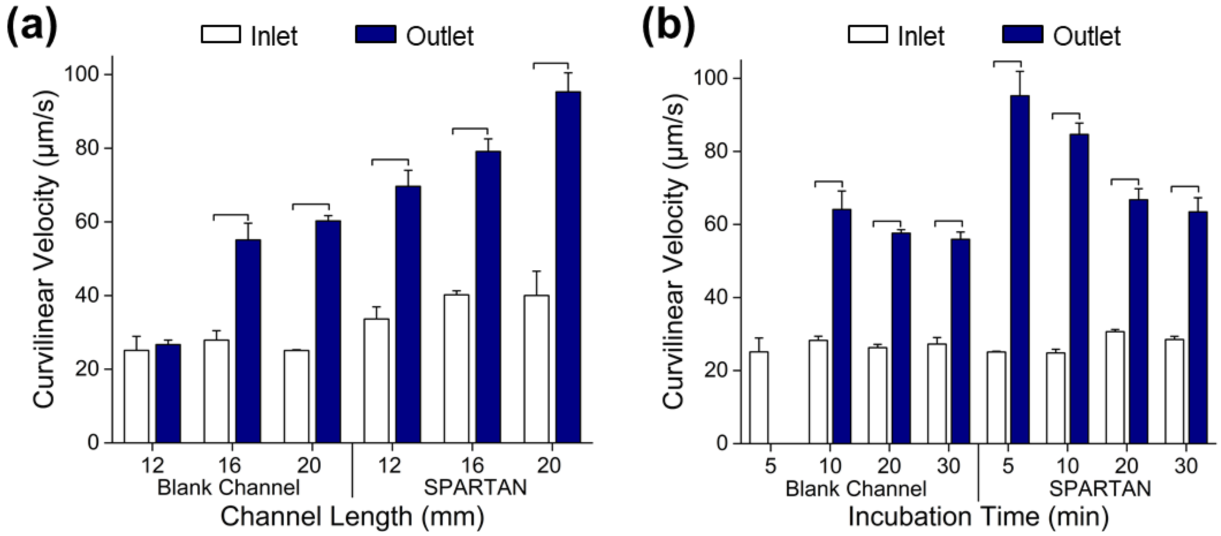

Figure S3: Experimental curvilinear velocities (VCL) for sperm in SPARTAN, compared to blank channels for various channel lengths and incubation times. (a) Outlet and inlet VCL values for the 30 x 26 µm periodicity device for lengths of 12, 16 and 20 mm, compared with the blank channel of similar lengths. Incubation at the outlet is 30 min. (b) Outlet and inlet VCL values for the 30 x 26 µm periodicity device with 16 mm of length for incubation times in the range 5-30 min, compared with the blank channel. Error bars show SEM. (n=10-1600, N=3). n denotes the number of sperm used in the analysis, and N represents the number of experiments performed in this assessment.

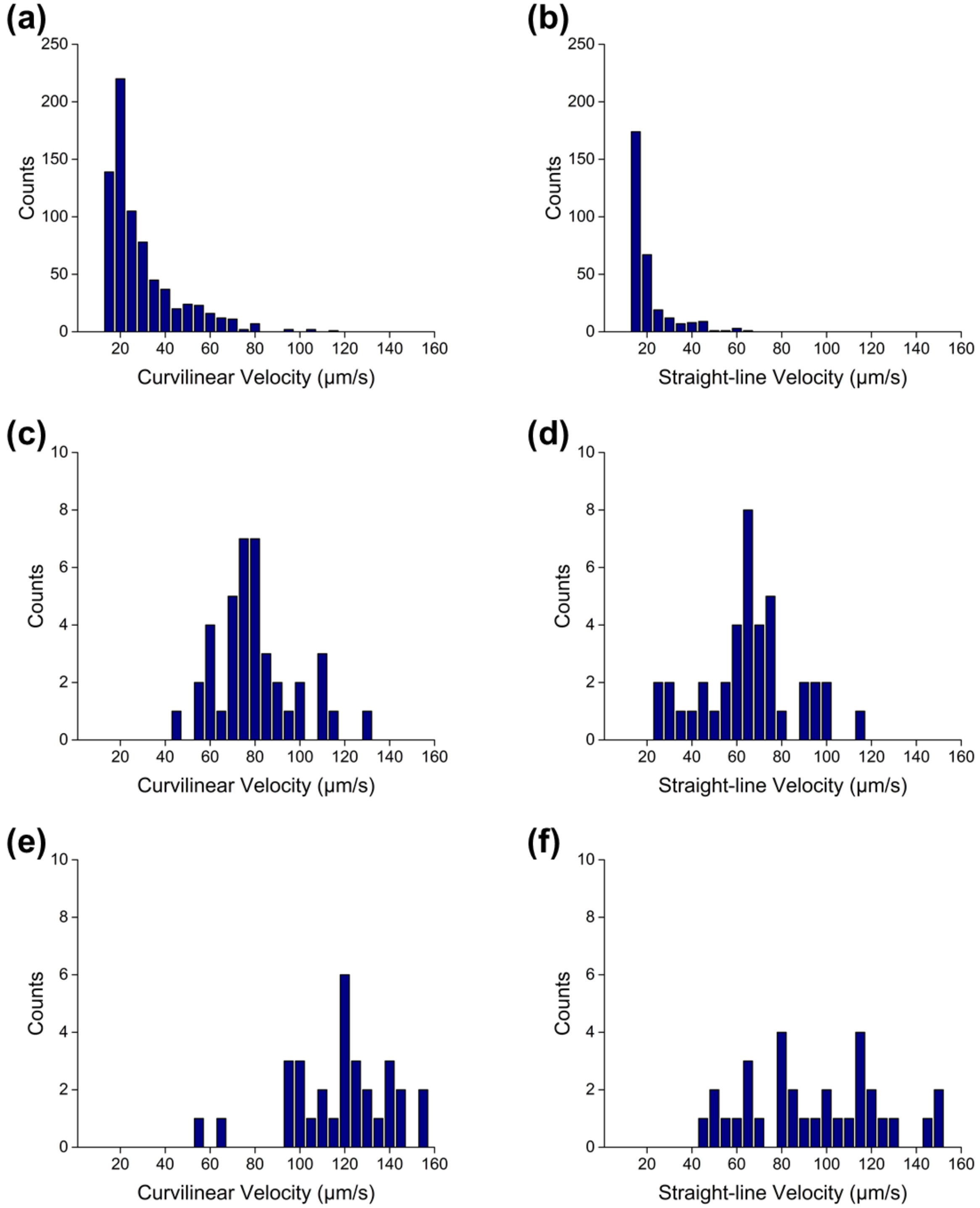

Figure S4: Sperm velocity distribution analysis. Velocity distributions shown as (a) curvilinear velocity (VCL), and (b) straight-line velocity (VSL) at the inlet; (c) VCL, and, (d) VSL within the pillar array; (e) VCL, and, (f) VSL at the outlet of SPARTAN after 5 minutes of incubation. All data was collected on the channels with 30x26 μm of pillar periodicity and 16 mm length.

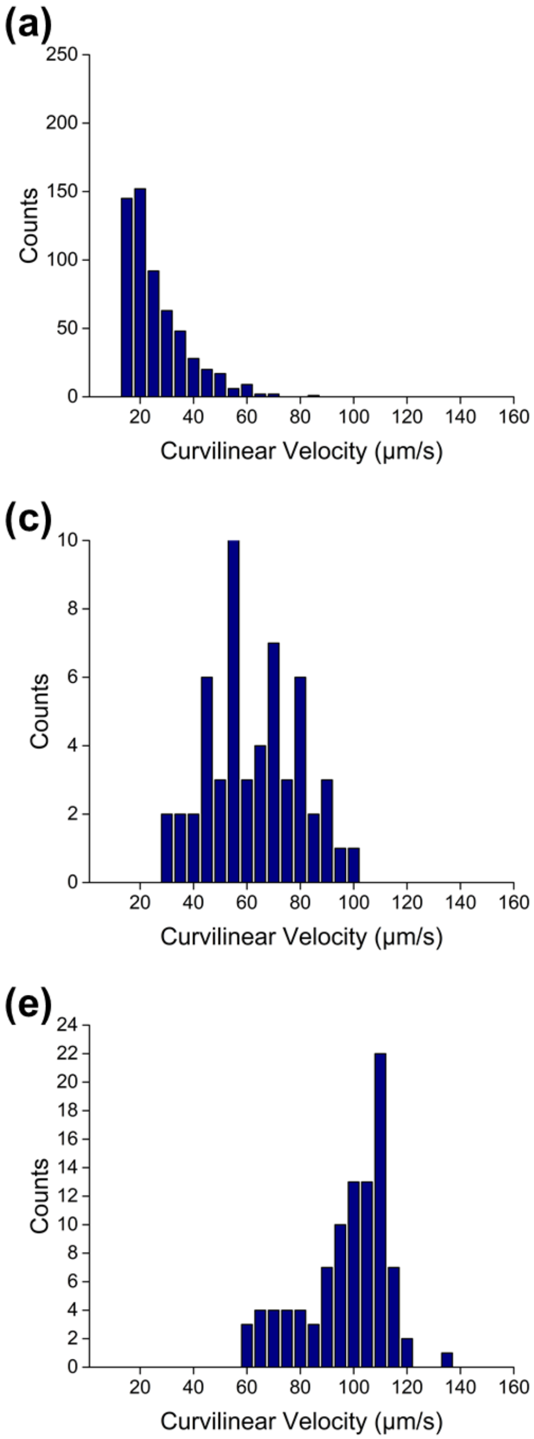

Figure S5: Sperm velocity distribution analysis. Velocity distributions shown as (a) curvilinear velocity (VCL), and, (b) straight-line velocity (VSL) at the inlet; (c) VCL and (d) VSL within the pillar array; (e) VCL and (f) VSL at the outlet of SPARTAN after 30 minutes of incubation. All data was collected on the channels with 30x26 μm of pillar periodicity and 16 mm length.

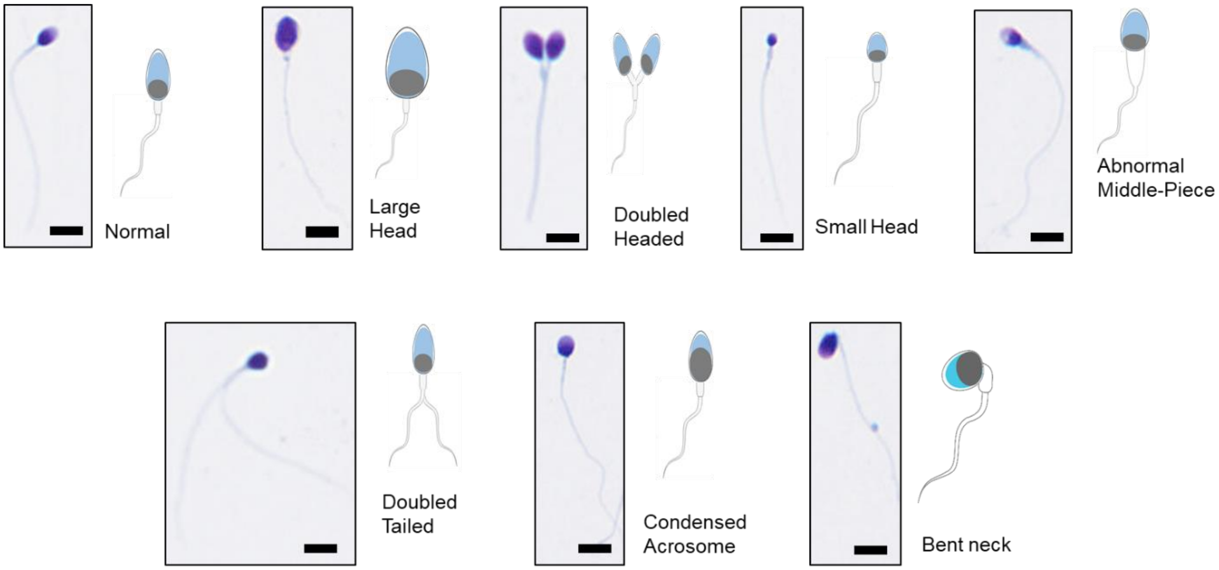

Figure S6. Different sperm morphologies observed in the sample analysis. Scale bars represent 10 µm.

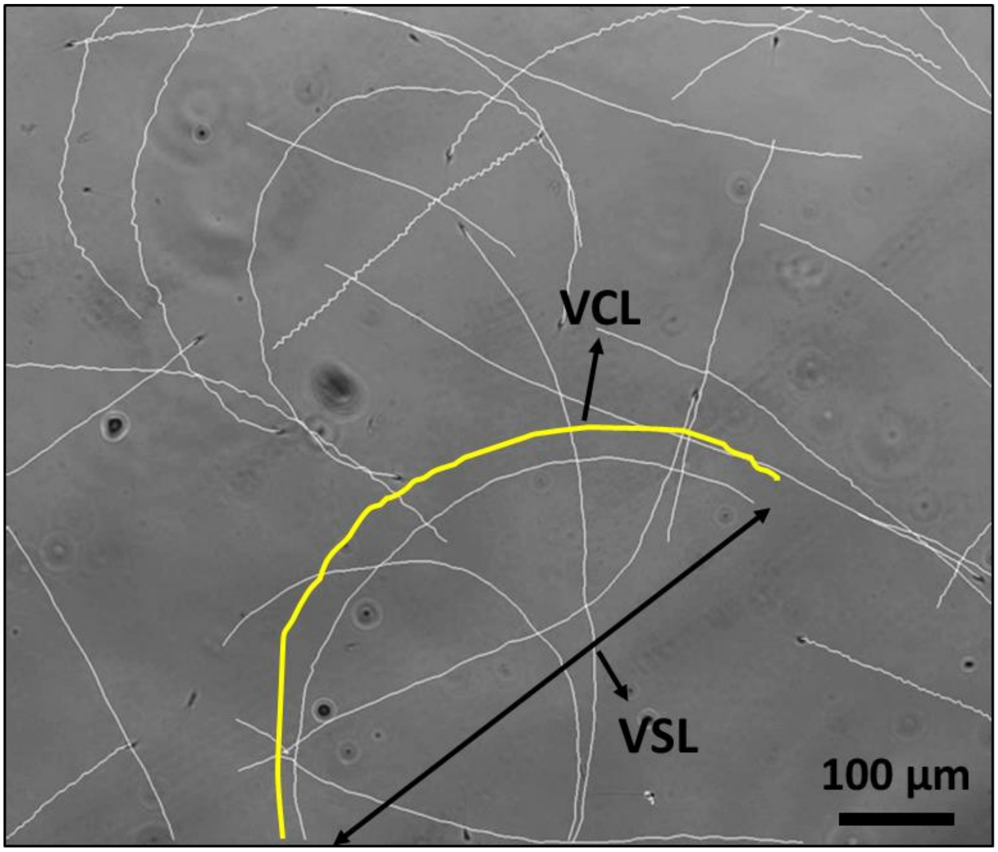

Figure S7: Illustration of Computer Assisted Sperm Analyzer (CASA) based motility measurements for sperm. Curvilinear velocity (VCL) refers to the distance that the sperm head covers during the observation time (i.e. the actual path, shown in yellow). Straight-line velocity (VSL) refers to the distance between the starting and ending points of a given sperm trajectory (shown in black).

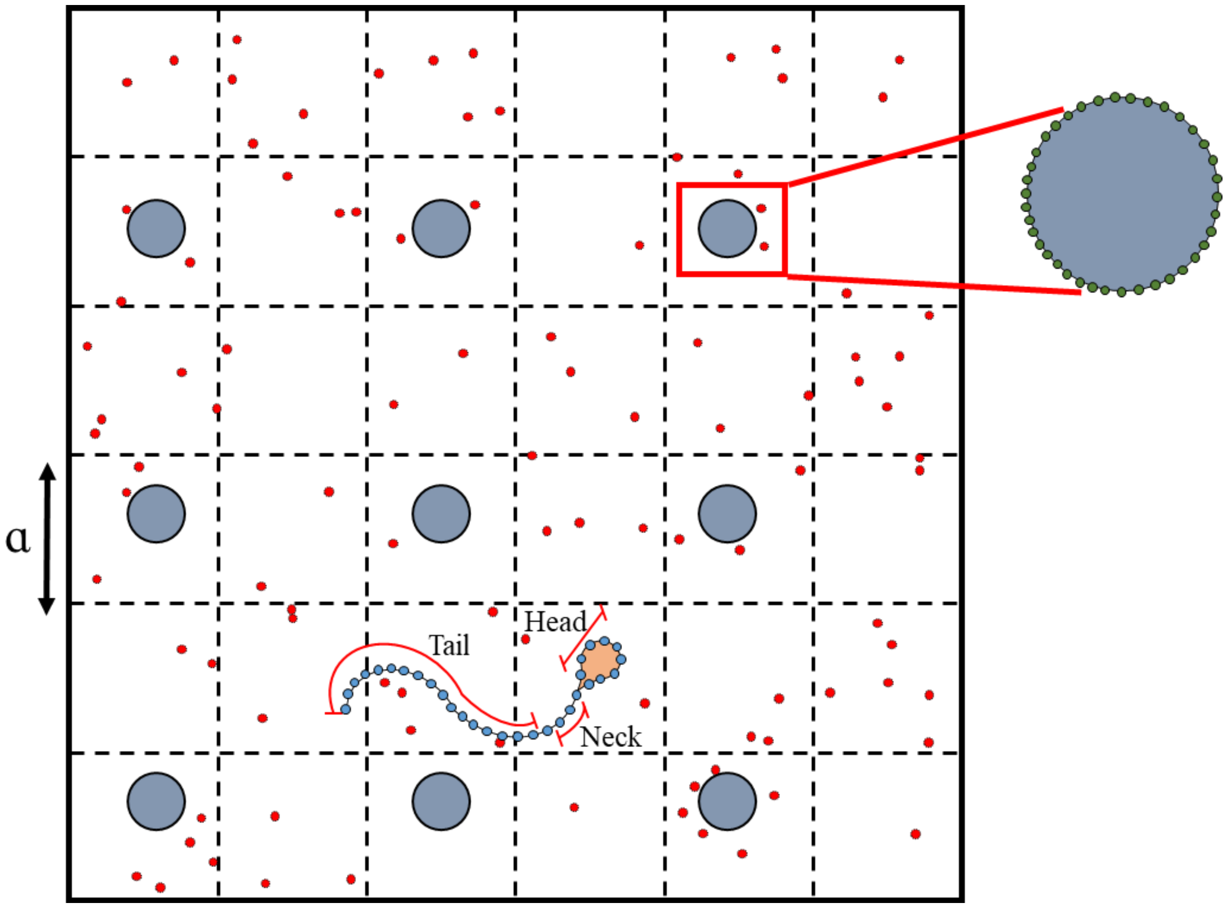

Figure S8: Illustration of the MPCD model geometry. MPCD dynamics consists of streaming and collision steps for the solvent particles shown in the figure. The collision cell size is a. The sperm cell is modeled as a bead-and-spring chain, governed by the sum of a spring and bending energies. Pillars are also modeled as series of connected beads (with distance *a* in between) that are immobile. The beads on the sperm backbone as well as the pillars both participate in the MPCD collision step. A velocity-Verlet scheme is used to integrate the equations of motion for the sperm backbone.

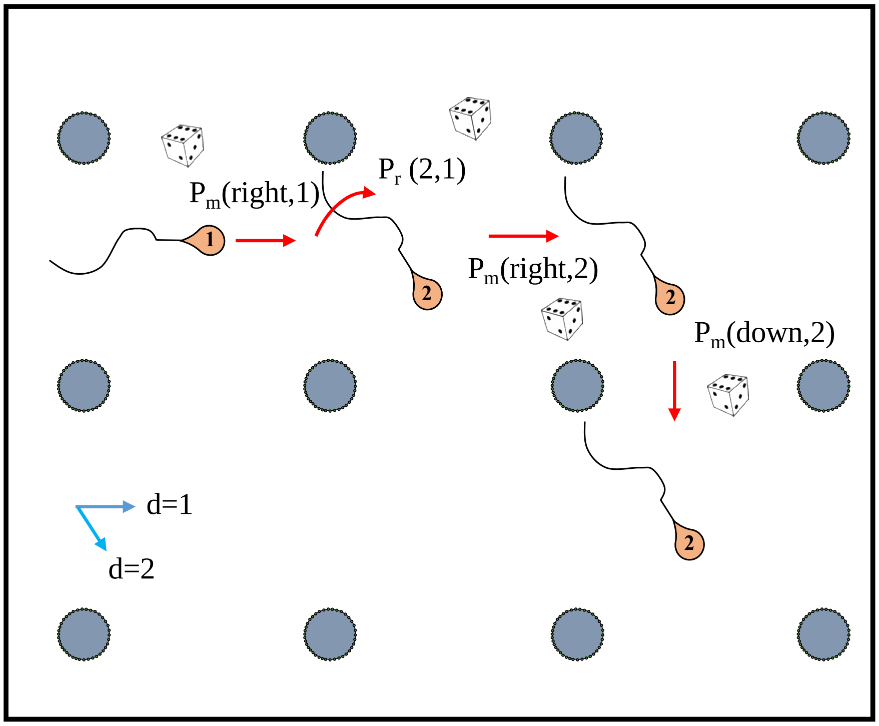

Figure S9: Illustration of the lattice model steps. P_m and P_r denote the movement and rotation probabilities, respectively. As an example, P_m(right,1) is the probability of going to the right, while pointing in the direction, d=1 (positive *x* axis); and P_r(2,1) is the probability of a transition from the rotational state *d=1* to *d=2*.

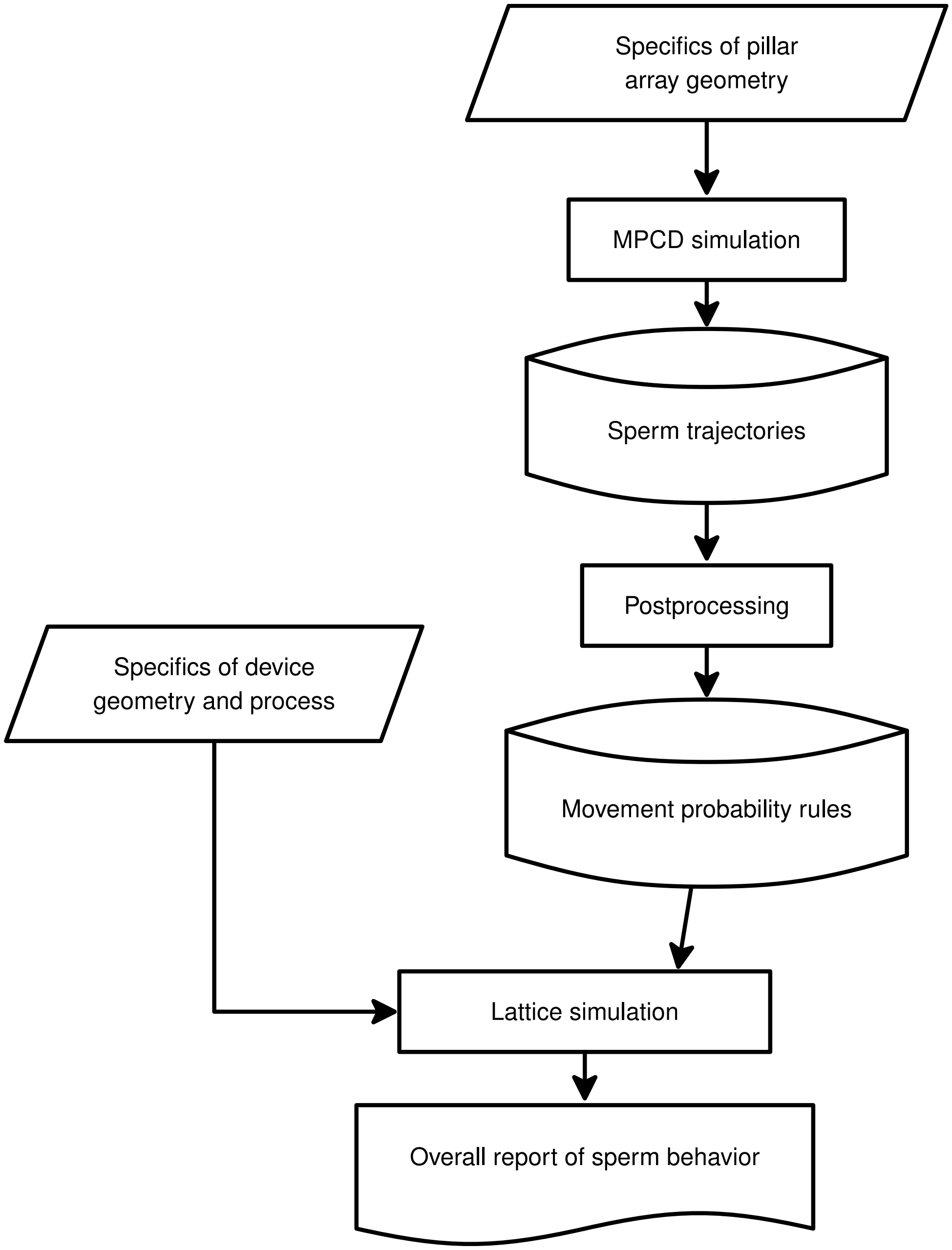

Figure S10: Overview of the modeling approach.

Supplementary Tables

Table S1: Sperm trajectory analysis results at the outlet of SPARTAN featuring devices for varying pillar periodicities. All data was collected on the channels with 12 mm length after 30 minutes of incubation time.

VSL (µm/s)						
**Pillar Periodicity (µm)**	**Inlet**			**Outlet**		
**18-26**	14.24171	15.12221	18.58142	59.65178	58.01342	50.77096
**22-22**	24.0078	36.35108	29.20984	68.16849	78.44618	60.33583
**22-26**	23.15562	21.94327	25.45671	61.43579	58.73681	54.9094
**26-26**	17.68628	17.33369	14.31462	59.61086	58.57181	48.84909
**30-26**	17.49171	15.75276	16.81709	73.6163	79.70698	75.02329

VCL (µm/s)						
**Pillar Periodicity (µm)**	**Inlet**			**Outlet**		
**18-26**	38.81319	44.43238	49.99136	76.40008	73.24052	67.77689
**22-22**	49.4766	46.20648	42.65729	80.75024	88.9149	69.36325
**22-26**	45.45779	40.56828	43.10548	77.17972	72.38605	72.13718
**26-26**	50.25471	47.76272	45.45975	72.15782	73.93568	64.29385
**30-26**	39.32616	40.65102	41.2763	85.51434	81.92055	88.42123

Table S2: Sperm trajectory analysis results at the outlets of blank and SPARTAN featuring channels of varying lengths. All data was collected on the channels with 30x26 µm of pillar periodicity after 30 minutes of incubation.

VSL (µm/s)							
	**Channel Length**	**Inlet**			**Outlet**		
**Blank Channel**	12 mm	13.47469	14.04084	16.69186	22.52191	22.52191	28.1309
	16 mm	12.63225	15.48276	14.08472	50.05127	50.05127	47.23323
	20 mm	18.64797	16.74561	11.67149	50.18563	56.66542	49.66454
**SPARTAN**	12 mm	15.12969	16.1066	20.41664	65.59264	59.97497	55.20185
	16 mm	32.14343	36.24918	36.27959	73.75194	72.97506	73.22389
	20 mm	26.02589	15.15575	15.2776	91.09541	95.94216	95.97881

VCL (µm/s)							
	**Channel Length**	**Inlet**			**Outlet**		
**Blank Channel**	12 mm	21.50894	29.07783	24.81407	25.86461	28.09558	26.11558
	16 mm	30.46226	25.37128	27.91699	56.82806	58.54937	49.92155
	20 mm	25.30066	25.05449	24.90315	60.97378	58.60388	61.23388
**SPARTAN**	12 mm	31.37584	32.15049	37.40739	73.98193	69.7243	65.32108
	16 mm	41.2726	39.10243	40.28225	82.0426	79.98945	75.40579
	20 mm	47.60286	35.582	36.88275	98.51319	89.33818	98.0774

Table S3: Sperm trajectory analysis results at the outlets of blank and SPARTAN featuring channels for varying incubation times. All data was collected on the channels with 30x26 µm of pillar periodicity and 16 mm length.

VSL (µm/s)							
	**Incubation Time**	**Inlet**			**Outlet**		
**Blank Channel**	5 min	18.64797	16.74561	11.67149	N/A	N/A	N/A
	10 min	13.47469	14.04084	16.69186	62.56895	62.26907	64.01635
	20 min	12.63225	15.48276	14.08472	51.28933	51.47179	49.09005
	30 min	18.64797	16.74561	11.67149	50.54927	46.11703	45.59873
**SPARTAN**	5 min	26.02589	25.15575	25.2776	96.2135	90.7923	95.70062
	10 min	23.23915	23.97936	22.38821	79.51119	76.74357	80.38137
	20 min	26.02589	25.15575	25.2776	67.3193	64.89477	69.11848
	30 min	32.14343	36.24918	36.27959	54.46119	54.48349	59.54484

VCL (µm/s)							
	**Incubation Time**	**Inlet**			**Outlet**		
**Blank Channel**	5 min	21.50894	29.07783	24.81407	N/A	N/A	N/A
	10 min	29.5142	27.28546	28.0076	60.44813	62.03413	69.86028
	20 min	26.5142	25.28546	27.0076	58.60648	56.67476	57.59814
	30 min	26.5142	29.28546	26.0076	54.61979	55.01417	58.23518
**SPARTAN**	5 min	25.30066	25.05449	24.90315	98.1614	87.5582	99.9288
	10 min	23.65959	25.31612	25.49513	82.52616	83.26225	88.21313
	20 min	30.56586	31.27657	30.12114	64.73057	70.23184	65.40537
	30 min	27.64024	28.47985	29.38253	61.09064	61.36073	67.90167

Table S4: Analysis of clinically relevant sperm characteristics. All data was collected on the channels with 30x26 µm of pillar periodicity and 16 mm length after 30 minutes of incubation time.

Normal Morphology (%)												
**Sample ID**	**Raw**			**Swim-up**			**Blank Channel**			**SPARTAN**		
**S1**	11	12	10	26	23	20	35	31	38	48	52	50
**S2**	12	14	9	25	23	28	42	39	45	55	50	51
**S3**	9	11	9	18	20	22	35	33	30	48	54	49
**S4**	14	15	17	25	23	23	41	37	44	51	48	55
**S5**	20	16	16	29	31	28	48	45	50	59	52	55

Nuclear Maturity (%)												
**Sample ID**	**Raw**			**Swim-up**			**Blank Channel**			**SPARTAN**		
**S1**	12	17	13	22	27	25	25	28	31	45	48	51
**S2**	11	9	9	19	23	20	31	29	33	36	35	34
**S3**	30	27	25	33	35	30	39	41	44	55	48	53
**S4**	15	13	14	27	25	25	41	37	42	42	46	48
**S5**	22	18	16	28	25	30	30	34	29	40	35	37

Table S5: Probabilities for the lattice model, for normal sperm swimming through the 30 x 26 µm pillar array.

**State** *n*	$P_r(n + 1, n)$	$P_r(n - 1, n)$	$P_m(\textbf{left}, \boldsymbol{n})$	$P_m(\textbf{up}, n)$	$P_m(\textbf{right}, n)$	$P_m(\textbf{down}, n)$
**0**	0.017986	0.051743	0.000000	0.006502	0.109574	0.000138
**1**	0.18949	0.143312	0.000000	0.000000	0.05414	0.025478
**2**	0.027669	0.024441	0.000000	0.000000	0.069864	0.071247
**3**	0.017903	0.023948	0.000000	0.000000	0.051616	0.078354
**4**	0.017962	0.003732	0.045079	0.000000	0.00005	0.087869
**5**	0.023525	0.033438	0.068922	0.000000	0.000000	0.063729
**6**	0.017986	0.051743	0.109574	0.000138	0.000000	0.006502
**7**	0.18949	0.143312	0.05414	0.025478	0.000000	0.000000
**8**	0.027669	0.024441	0.069864	0.071247	0.000000	0.000000
**9**	0.017903	0.023948	0.051616	0.078354	0.000000	0.000000
**10**	0.017962	0.003732	0.00005	0.087869	0.045079	0.000000
**11**	0.023525	0.033438	0.000000	0.063729	0.068922	0.000000

Table S6: Probabilities for the lattice model, for the sperm with a 18° head bend swimming through the 30 x 26 μm pillar array.

**State** *n*	$P_r(n+1, n)$	$P_r(n-1, n)$	P_m(**left**, n)	P_m(**up**, n)	P_m(**right**, n)	P_m(**down**, n)
0	0.004691	0.041946	0.000000	0.008003	0.096403	0.000828
1	0.050683	0.102153	0.000000	0.000000	0.058036	0.034664
2	0.00897	0.056848	0.000000	0.000000	0.070304	0.064984
3	0.039471	0.085735	0.000000	0.000000	0.026069	0.092712
4	0.005098	0.062526	0.002601	0.000000	0.000000	0.101124
5	0.033138	0.101217	0.034491	0.000000	0.000000	0.066952
6	0.00712	0.092068	0.057129	0.000000	0.000000	0.089088
7	0.004691	0.041946	0.096403	0.000828	0.000000	0.008003
8	0.050683	0.102153	0.058036	0.034664	0.000000	0.000000
9	0.00897	0.056848	0.070304	0.064984	0.000000	0.000000
10	0.039471	0.085735	0.026069	0.092712	0.000000	0.000000
11	0.005098	0.062526	0.000000	0.101124	0.002601	0.000000
12	0.033138	0.101217	0.000000	0.066952	0.034491	0.000000
13	0.00712	0.092068	0.000000	0.089088	0.057129	0.000000

Table S7: Probabilities for the lattice model, for sperm with a 3 times larger head swimming through the 30 x 26 µm pillar array.

**State** *n*	$P_r(n+1, n)$	$P_r(n-1, n)$	$P_m(\textbf{left}, n)$	$P_m(\textbf{up}, n)$	$P_m(\textbf{right}, n)$	$P_m(\textbf{down}, n)$
0	0.01191	0.012928	0.000539	0.001017	0.073977	0.000898
1	0.057131	0.024724	0.001257	0.000978	0.058528	0.029334
2	0.031656	0.072559	0.000534	0.001423	0.00907	0.079139
3	0.089185	0.096361	0.000000	0.001025	0.002563	0.082522
4	0.037699	0.042711	0.000654	0.001743	0.000000	0.068207
5	0.010966	0.014597	0.040087	0.001162	0.00138	0.049020
6	0.01191	0.012928	0.073977	0.000898	0.000539	0.001017
7	0.057131	0.024724	0.058528	0.029334	0.001257	0.000978
8	0.031656	0.072559	0.00907	0.079139	0.000534	0.001423
9	0.089185	0.096361	0.002563	0.082522	0.000000	0.001025
10	0.037699	0.042711	0.000000	0.068207	0.000654	0.001743
11	0.010966	0.014597	0.00138	0.04902	0.040087	0.001162

Supplementary Videos

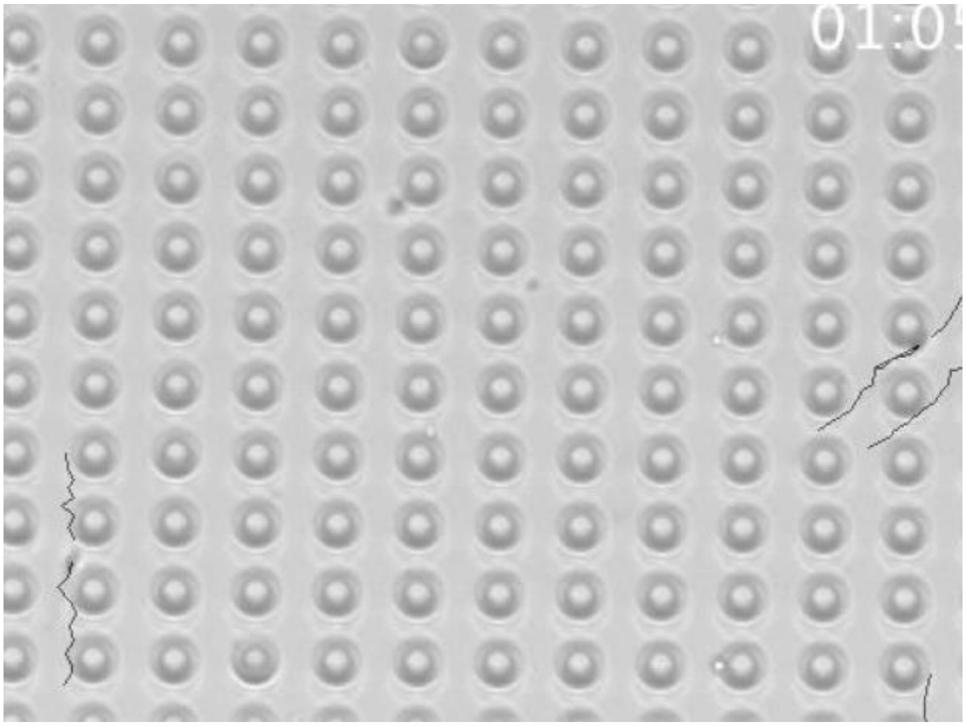

Video S1: Experimental sperm trajectory movie for the 18-26 µm array periodicity channel.

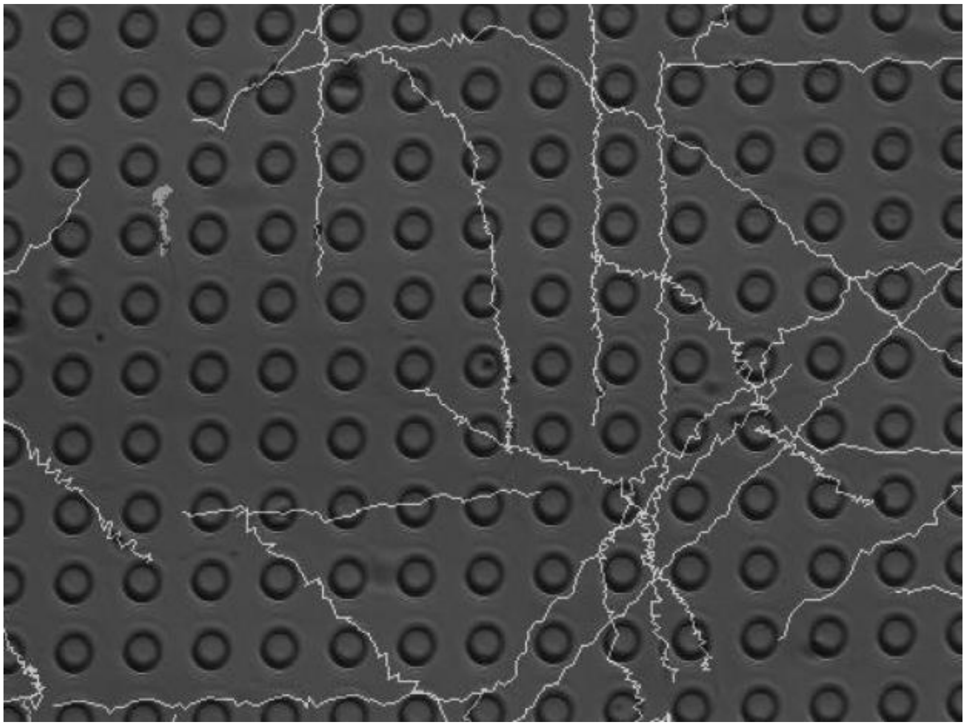

Video S2: Experimental sperm trajectory movie for the 22-22 μm array periodicity channel.

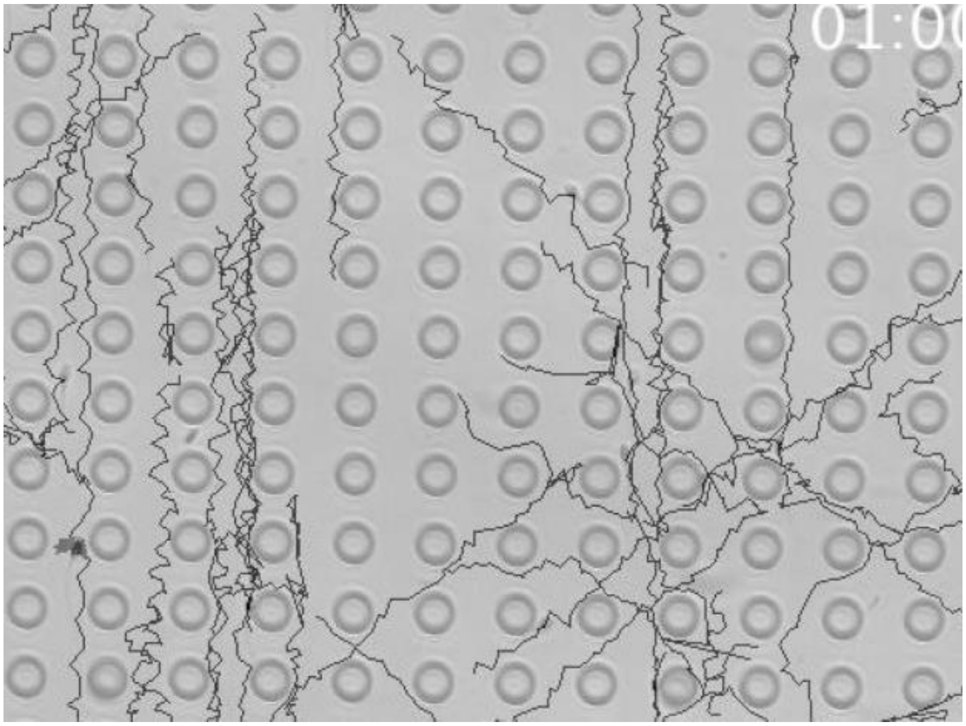

Video S3: Experimental sperm trajectory movie for the 22-26 µm array periodicity channel.

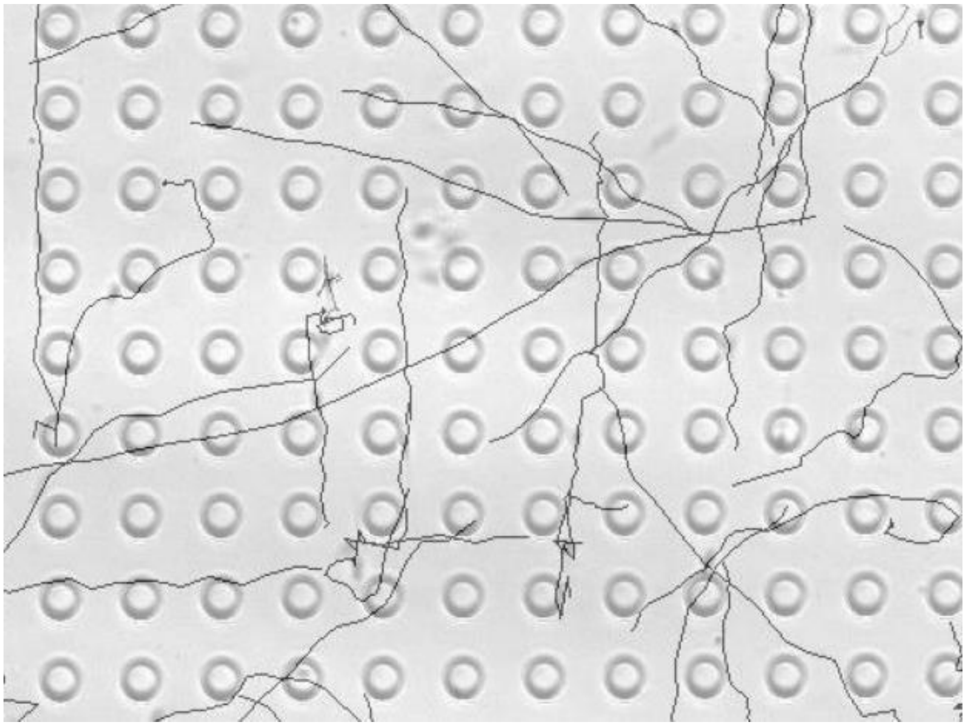

Video S4: Experimental sperm trajectory movie for the 26-26 μm array periodicity channel.

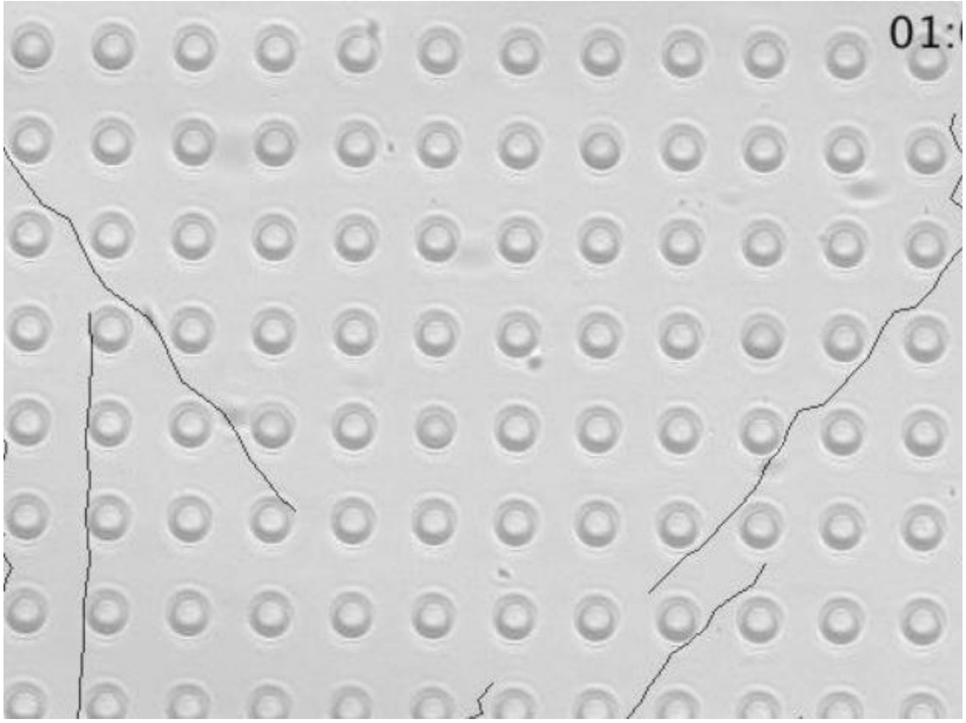

Video S5: Experimental sperm trajectory movie for the 30-26 µm array periodicity channel.

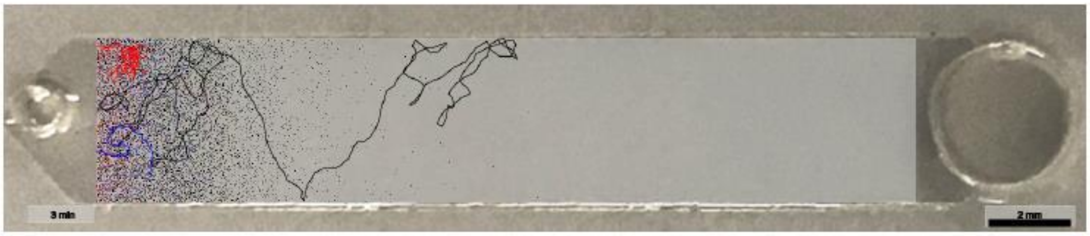

Video S6: Simulation of an ensemble of sperm with normal morphology (black, n=20000), big heads (blue, n=2000), and bent necks (red, n=2000) using the lattice model for channel with a 20X4 mm post array. One representative sperm of each set is chosen to trace its path, while the rest are rendered as their single point. The curving (amorphous sperm) and circular (bent-neck sperm) trajectories of sperm through the pillar array is in stark contrast to the observed linear trajectory of morphological normal sperm.

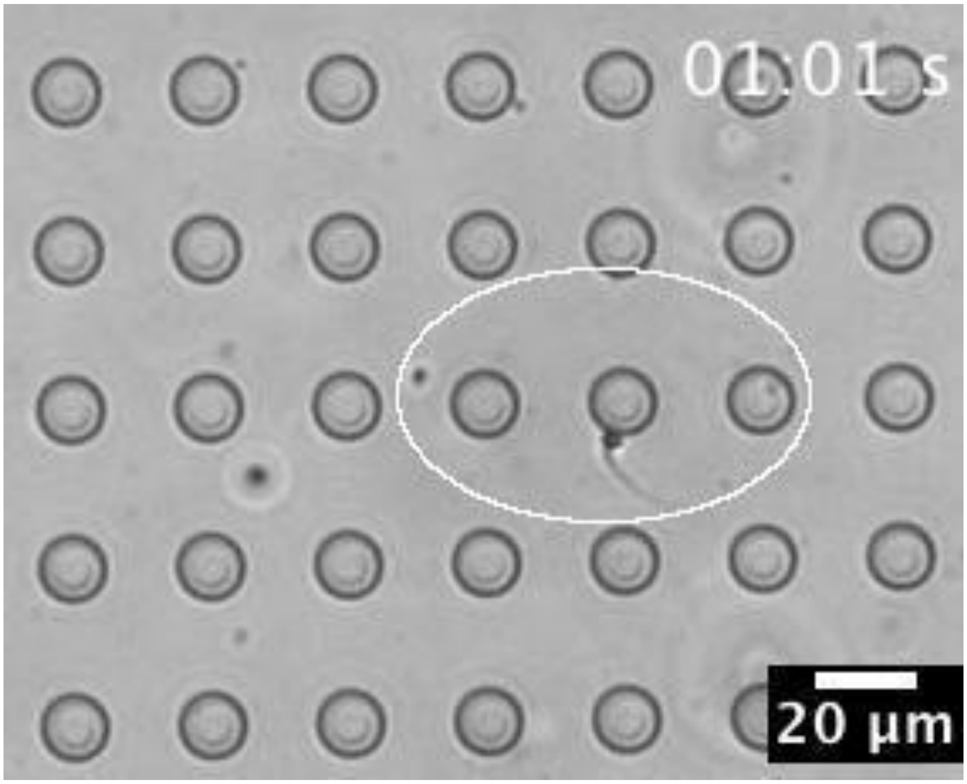

Video S7: Sperm with bent-neck abnormal morphology stuck in the pillar array of SPARTAN.

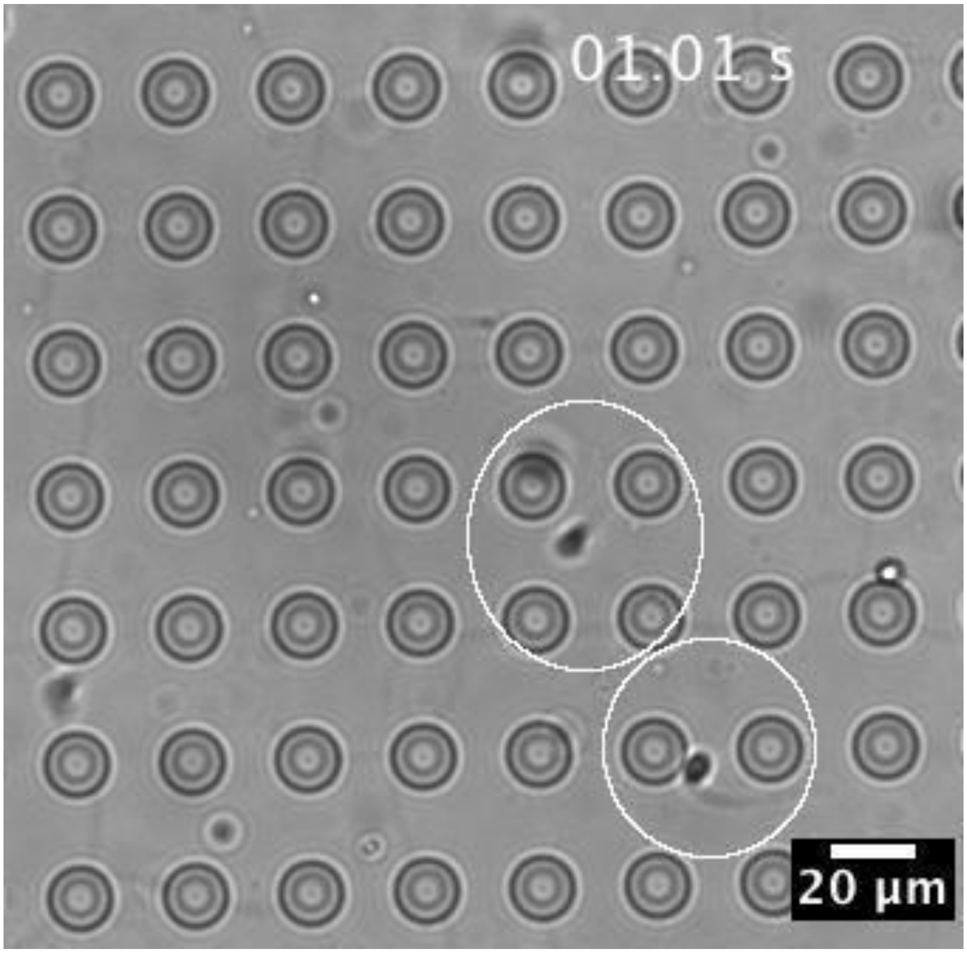

Video S8: Sperm with large and bent-neck abnormal morphology stuck in the pillar array of SPARTAN.

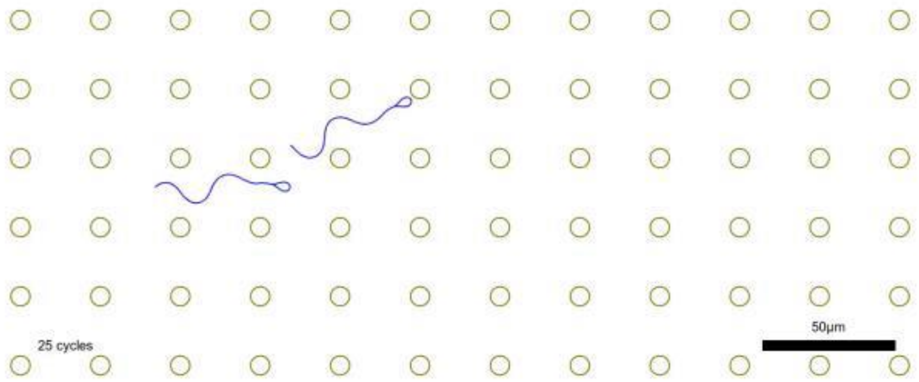

Video S9: Simulation of sperm swimming through a 30 X 26 μm post array. The sperm move in a highly progressive manner through the array, with direction changes at random intervals.

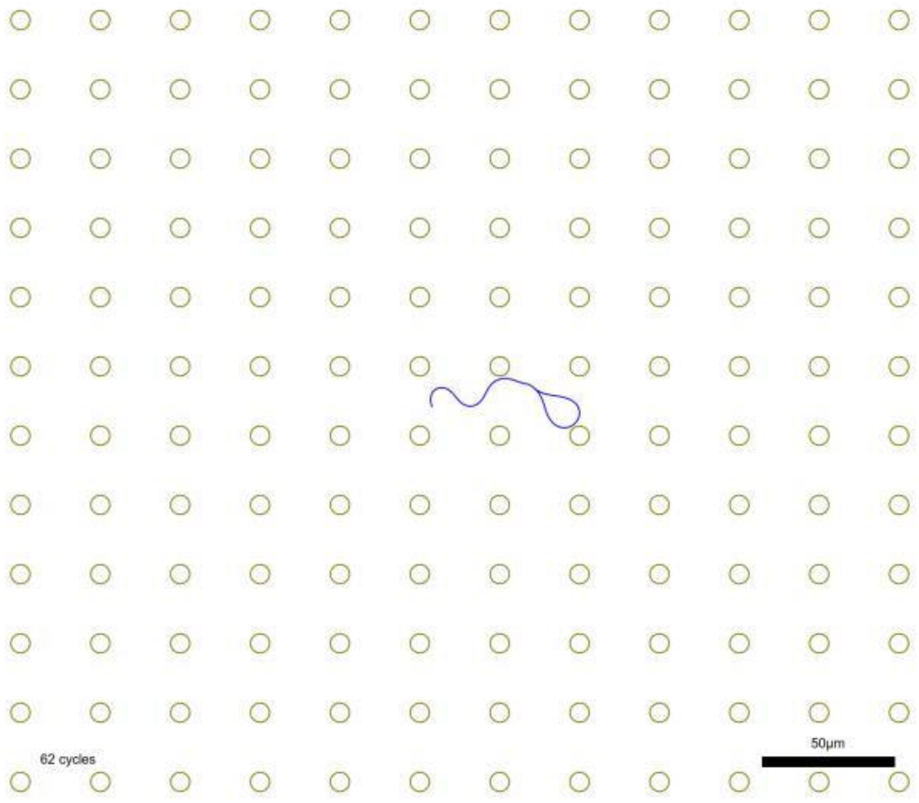

Video S10: Simulation of sperm with a large head (3X normal) swimming through a 30X26 μm post array. The larger head causes the sperm to get partially stuck and make sharp turns, reducing its ability to effectively traverse the post array.

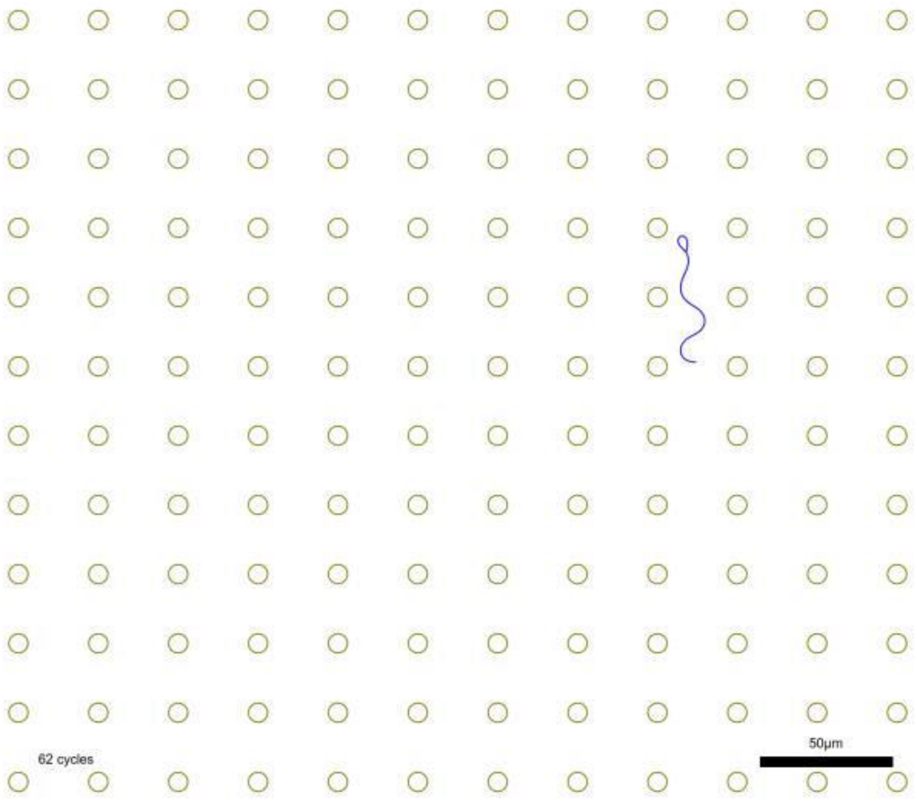

Video S11: Simulation of sperm with a bent neck (18 degrees) swimming through a 30X26 μm post array. Despite the relatively subtle bend, the sperm stays confined in a circle, preventing it from leaving the array within the duration of the experiment.
